# How to Keep the Balance between Red and Processed Meat Intake and Physical Activity Regarding Mortality: A Dose-Response Meta-Analysis

**DOI:** 10.3390/nu15153373

**Published:** 2023-07-29

**Authors:** Yi Wu, Maoqing Wang, Zhiping Long, Jingyu Ye, Yukun Cao, Bing Pei, Yu Gao, Yue Yu, Zhen Han, Fan Wang, Yashuang Zhao

**Affiliations:** 1 Department of Epidemiology, School of Public Health, Harbin Medical University, 157 Baojian Road, Nangang District, Harbin 150028, China; wywxg51@163.com (Y.W.); longzhiping0403@163.com (Z.L.); yejingyu2016@163.com (J.Y.); cyk1476612544@163.com (Y.C.); peibing980319@163.com (B.P.); gaoyu@hrbmu.edu.cn (Y.G.); yuyue1997@126.com (Y.Y.); 2020020160@hrbmu.edu.cn (Z.H.); 2 National Key Disciplines of Nutrition and Food Hygiene, Department of Nutrition and Food Hygiene, School of Public Health, Harbin Medical University, 157 Baojian Rd., Nangang District, Harbin 150028, China; wang_maoqing@126.com; 3 NHC Key Laboratory of Etiology and Epidemiology, Harbin 150028, China

**Keywords:** red meat, processed meat, walking, muscle-strengthening activities, all-cause mortality, cardiovascular disease mortality, cancer mortality

## Abstract

Background: Non-communicable diseases have become a major threat to public health, with cardiovascular diseases (CVDs) and cancer being the top two causes of death each year. Objective: Our objective is to evaluate the balanced association between the effect of red and processed meat intake on the risk of death and the effect of physical activity on the risk of mortality, where the risk of death includes all causes, CVDs, and cancers. Methods: We searched electronic databases, including PubMed, ISI Web of Science, Embase, and the Cochrane Library, for prospective studies reporting risk estimates for the association between the intake of red and processed meat, walking, and muscle-strengthening activity (MSA) and the risk of mortality from all causes, CVDs, and cancer. We extracted fully adjusted effect estimates from original studies and performed a summary analysis using the fixed and random-effect models. Results: A conventional meta-analysis showed that red meat and processed meat were positively associated with the risk of mortality, and daily steps and MSA were negatively associated with the risk of death. Further analysis of the dose–response relationship showed that a risk reduction (20%) from 39.5 min/week of MSA or 4100 steps/d was equivalent to an increased risk of all-cause mortality from a daily intake of 103.4 g/d of red meat or 50 g/d of processed meat. The risk was further decreased as the number of steps per day increased, but the risk reversed when the MSA exceeded the threshold (39.5 min/week). Conclusions: Adherence to physical activity is an effective way to reduce the risk of mortality due to meat intake. However, the total intake of red meat and processed meat should be controlled, especially the latter. Walking is recommended as the main daily physical activity of choice, while MSAs are preferred when time is limited, but it should be noted that longer MSAs do not provide additional benefits.

## 1. Introduction

Non-communicable diseases are one of the major challenges to public health, causing 71% of global deaths each year. Cardiovascular diseases (CVDs) are the leading cause of death from non-communicable diseases, accounting for 17.9 million deaths annually, followed by cancer (9.3 million) [1].

Unhealthy diets and physical activity are major risks and protective factors for chronic diseases and associated mortality, respectively [1]. Meat and its products are the main source of dietary protein for humans. Red and processed meat consumption remains high in developed countries like the USA and the UK [2,3], and it is increasing at a rate of 5–6% per year in developing countries, with the majority of consumption being red meat [4]. However, according to estimates from the Global Burden of Disease Study, approximately 813,307 and 231,091 people died from CVDs and cancers, respectively, in 2019 because of excessive red and processed meat consumption, with the majority of these deaths occurring in high- and middle-income countries. Physical activity can generally be categorized as aerobic or anaerobic, with walking being the simplest form of aerobic exercise. Anaerobic muscle-strengthening activities (MSAs) are defined as “activities that use equipment that primarily involves upper body movement and muscle strengthening”, including push-ups, bench-presses, sit-ups, and weight-lifting activities. The benefits of walking and MSAs contribute not only to lowering incidents of CVDs, diabetes, obesity, and cancers but also to lowering the mortality rate from multiple chronic conditions [5,6]. Findings from 15 international cohorts suggested that subjects who walked 10,901 steps per day had a 53% lower risk of all-cause mortality compared with 3553 steps [7]. Another study showed that MSAs over 60–150 min per week were associated with a lower mortality risk from CVDs by 20%–25%, cancer by 15–20%, and overall mortality by 20–25% [8].

Although numerous reports have revealed the harmful effects of excessive meat intake on mortality risk, few studies provide any insight into how to choose between red and processed meat. Even less evidence is available on how one should choose between walking and MSAs. From the perspective of individual prevention, it is crucial to accurately balance the mortality risk caused by exposure to risk and protective factors according to dose, but, to the best of our knowledge, this has never been evaluated. Therefore, this study aims to reveal a balanced scale by assessing the dose–response relationship between meat intake, daily steps, and MSAs on mortality outcomes based on a systematic review and dose–response meta-analysis.

## 2. Methods

Findings from this systematic review and meta-analysis were reported based on the Preferred Reporting Items for Systematic Review and Meta-Analyses (PRISMA) guidelines. The review protocol is registered with the PROSPERO database (CRD42022320178).

### 2.1. Search Strategy

**Figure 1:**
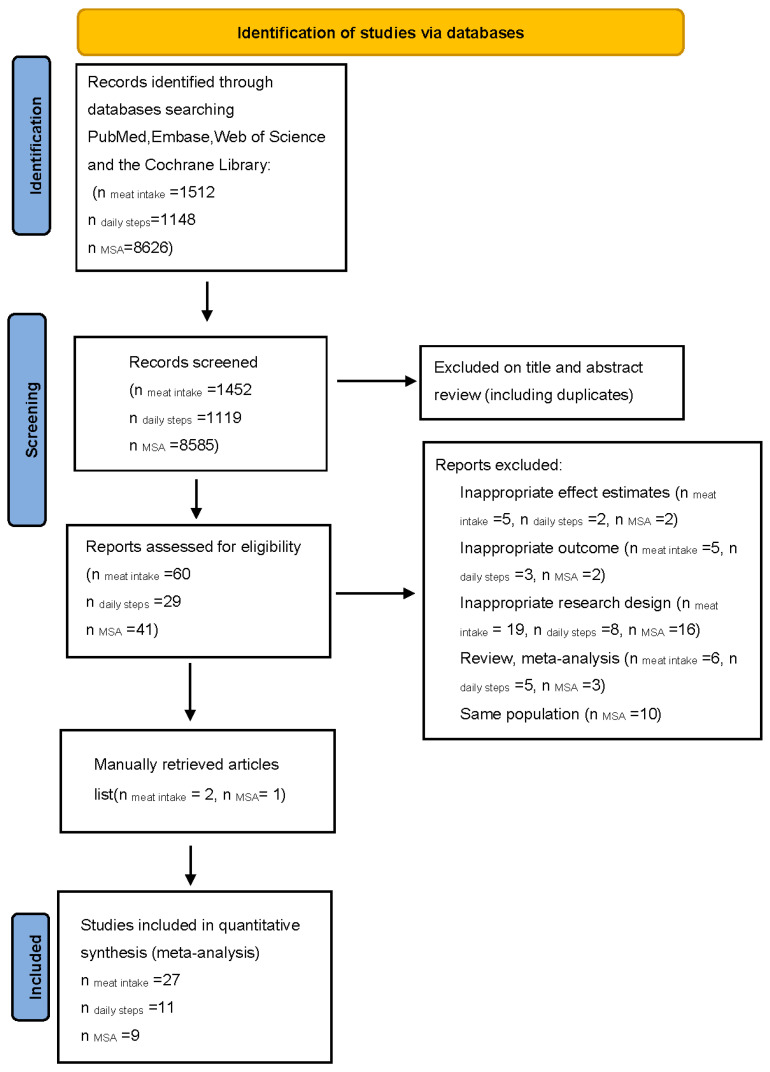
Preferred Reporting Items for Systematic Reviews and Meta-Analyses (PRISMA) flow diagram for search strategy.

We conducted a systematic search of all articles in English published up to 31 December 2021 in online databases, including PubMed, Web of Science, Embase, and the Cochrane Library. The reference lists of selected papers and recent reviews were cross-checked manually to identify any articles that might have been missed. Details of the study’s selection process are shown in Figure 1. Table S1 provides the information on the search terms.

### 2.2. Study Selection and Eligibility Criteria

Published studies were included if they were observational prospective studies conducted on human adults (aged ≥18 years) and with reported effect sizes, including hazard ratios (HRs) or relative risks (RRs) with corresponding 95% confidence intervals (95% CIs). All outcomes were classified based on the World Health Organization’s ICD-10 (International Classification of Diseases, 10th revision) or ICD-9 (9th revision) in original studies. If the same data set was published in more than one publication, the most recent publication or that with the largest number of participants was included. The inclusion criteria were (1) studies that analyzed the association between the intake of red meat, processed meat, or the mixed consumption of both as the exposure and mortality from all causes, CVDs, or cancers as the outcome; (2) studies that analyzed the association between MSAs and mortality from all causes, CVDs, or cancers; (3) studies that analyzed the association between daily steps and mortality from all causes, CVDs, or cancers. The exclusion criteria were (1) letters, comments, reviews, meta-analyses, and ecological studies; (2) studies performed on children or adolescents or patients with end-stage cancer or critical illness; (3) studies with insufficient data (e.g., exposure or outcome data).

### 2.3. Data Extraction

Two authors (Y.W. and Y.C.) independently extracted data from eligible studies by using a standard data extraction form; disagreements were resolved by consensus between the two reviewers or in group meetings. In group meetings, the two reviewers discuss and compare each other’s extraction results, and all the members involved in the data extraction stage (Y.W., Y.C., B.P. and J.Y.) reach a consensus based on the discussed results.

From each eligible article, we extracted the first author’s name, year of publication, population ethnicity, study design, age range, health status at the start of the study, sex, cohort size, number of outcomes, follow-up time, exposure, exposure assessment methods, outcomes, outcome assessment methods, comparison categories, HRs, RRs, 95% CIs, and variables adjusted for in the analysis. If the included studies reported both crude and multivariable-adjusted effect estimates, the most fully adjusted outcomes were used in the present meta-analysis.

### 2.4. Risk of Bias Assessment and GRADE Assessment

The Newcastle–Ottawa scale (NOS) was used to assess the quality of included studies. The NOS was adapted to evaluate the selection, comparability, and outcome/exposure of the individual study [9]. A star was awarded for every quality item, with a maximum of nine stars for the highest quality. Thus, 0–3, 4–6, and 7–9 stars were considered poor, fair, and good quality, respectively (Table S4). The certainty of the evidence was assessed using the GRADE (Grading of Recommendations Assessment, Development, and Evaluation) approach for each outcome [10]. According to GRADE, observational studies start at low certainty and may be downgraded for risk of bias, inconsistency, indirectness, imprecision, or publication bias and may be upgraded for a large effect, if suspected biases work against the observed direction of the effect or for a dose–response gradient. All assessments were conducted independently by two reviewers (Y.W. and Y.C.), and disagreements were resolved by consensus between the two reviewers or in a group meeting.

### 2.5. Data Synthesis and Statistical Analysis

In the present meta-analysis, the HRs and 95% CIs were considered the effect size for all studies, and the RRs were deemed equivalent to the HRs. The HRs and 95% CIs for comparisons of the highest versus lowest categories of meat intake, MSAs, and daily steps were used to calculate log HRs with standard errors for the primary analyses. Heterogeneity between studies was evaluated with *Q* and *I*^2^ statistics. For the *Q* statistic, a *p* < 0.1 was considered significant. The *I^2^* value was used to assess the amount of heterogeneity between studies, with an *I*^2^ ≥ 50 being of high heterogeneity, and a random effect model (Paule and Mandel) was chosen. An *I*^2^ < 50 was considered to show low heterogeneity, and a fixed effect model (Paule and Mandel) was chosen. A funnel plot was generated to assess the potential publication bias, and Egger’s linear regression and Beeg’s rank test were applied to test the symmetry. We also conducted sensitivity analyses to investigate the stability of the results, in which each prospective cohort study was excluded in turn to examine the influence of that study on the overall estimate. Subgroup analyses were conducted to investigate the source of heterogeneity.

In addition, the linear trends and nonlinear dose–response trends were both examined to describe the association between red and processed meat intake, MSAs, daily steps, and mortality outcomes using a restricted cubic spline model of four-section fixed-percentile (5%, 35%, 65%, and 95%) exposure distribution to evaluate the association. For this purpose, exposure dose, the number of cases, person-years, and adjusted HRs with 95% CIs across three or more quantitative categories in each study were extracted. If only the total number of cases or person-years was reported, the distribution of cases or person-years was estimated using the total number of cases and person-years [11]. If the total number of person-years was not reported, we approximated it by multiplying the total number of participants by the median or mean of the follow-up period. For studies reporting exposure in each category as a range, we considered the midpoint of the upper and lower bounds. Open-ended categories were considered to have the same widths as the closest category. Finally, the balanced scale was performed based on the results of dose–response relationships by calculating the inverse equilibrium of the HR suffered because of meat intake and the exposed dose of physical activity. Statistical analyses were conducted using STATA version 16.0 and R version 4.1.1.

## 3. Results

Table 1, Table 2 and Table 3 and Tables S2 and S3 show the characteristics of the studies included. The study quality was, in general, high because the vast majority of studies were in the group with 7–9 stars (Table S4). The respective means (medians) were 7.7 and 8 for meat intake and mortality, 8.18 and 8 for daily steps and all-cause mortality, and 7 and 7 for MSA and mortality.

### 3.1. Meta-Analysis of Meat Intake, Physical Activity, and Mortality Risk

Overall, 11,286 articles were identified in the initial search. Following the exclusion of duplicates and those that did not meet the inclusion criteria, 130 full-text articles of potentially relevant studies were identified. In total, 76 records were excluded because they were review and meta-analysis studies (*n* = 14); the exposure or outcome was not of interest (*n* = 10); the studies had insufficient data (*n* = 9); or the studies were on an unhealthy population (*n* = 43). We further excluded 10 records because of multiple publications from the same population cohorts. By contrast, three studies were added after a manual search and review of the reference lists. Finally, 47 articles were included (Figure 1).

Of the 47 articles, 27 reported the effect sizes for meat intake with 5,893,740 participants and 435,880 deaths, 11 reported the effect sizes of daily steps with 173,041 participants and 46,159 deaths, and 9 reported the effect sizes of MSAs with 1,023,245 participants and 103,211 deaths. Of these publications, 40 reported the effect sizes of all-cause mortality, 23 of CVD mortality, and 27 of cancer mortality.

**Figure 2:**
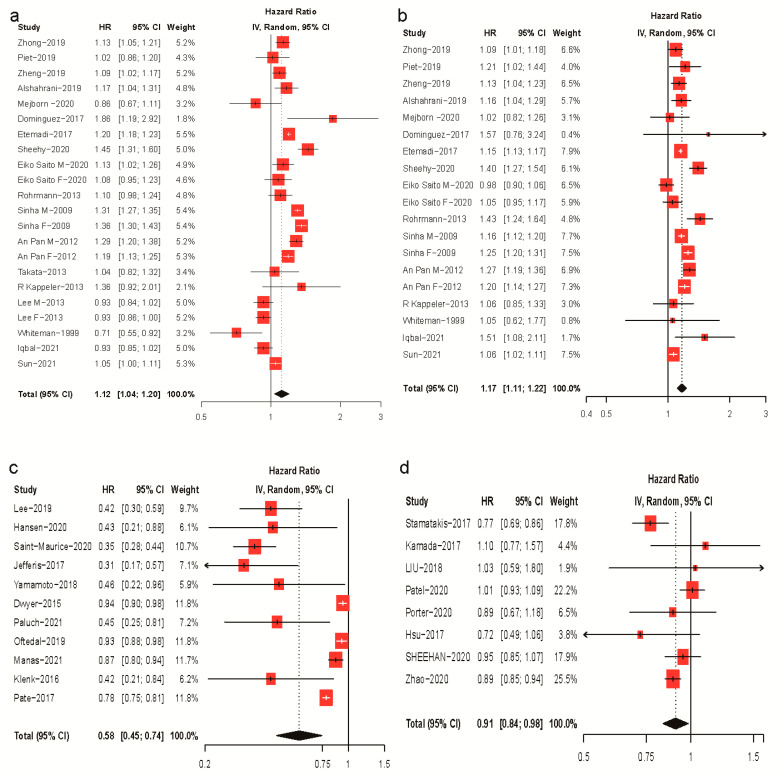
Forest plots of meta-analyses of red and processed meat intake, daily steps, and MSA and all-cause mortality. (**a**) Red meat intake and all-cause mortality; (**b**) processed meat intake and all-cause mortality; (**c**) daily steps and all-cause mortality; (**d**) MSA and all-cause mortality.

In comparisons of the highest versus lowest categories, the daily intake of red meat (18 papers [12,13,14,15,16,17,18,19,20,21,22,23,24,25,26,27,28,29]) and processed meat (16 papers [12,13,14,15,16,17,19,20,21,22,23,24,25,27,28,29]) was associated with a 12% (HR: 1.12; 95% CI: 1.04–1.20) and 17% (HR: 0.77; 95% CI 0.61–0.98) increased risk of all-cause mortality, respectively (Figure 2). Daily steps (eleven papers [30,31,32,33,34,35,36,37,38,39,40]) and MSAs (eight papers [41,42,43,44,45,46,47,48]) were associated with a 42% (HR: 0.58; 95% CI: 0.45–0.74) and 9% (HR: 0.91; 95% CI: 0.84–0.98) lower risk of all-cause mortality, respectively (Figure 2). Substantial heterogeneity was observed in the included studies (*I*^2^ = 92%, *p* < 0.01 for red meat; *I*^2^ = 80%, *p* < 0.01 for processed meat; *I*^2^ = 93%, *p* < 0.01 for daily steps; *I^2^* = 62%, *p* < 0.01 for MSAs). No evidence of publication bias was found in the analyses of red meat (Egger’s test, *p* = 0.069; Begg’s test, *p* = 0.430), processed meat (*p* = 0.638; *p* = 0.576), or MSA (*p* = 0.994; *p* = 0.902). Little evidence of publication bias was observed in the daily steps analysis (Egger’s test, *p* = 0.040; Begg’s test, *p* = 0.815) (Table S10).

Subgroup comparisons and sensitivity analyses indicated that, for all-cause mortality risks, there was no significant difference between red and processed meat (*p* = 0.34), whereas walking was more effective than MSA (*p* < 0.01). Insufficient data were available for analyses of CVDs and cancer mortality regarding walking. Detailed results are shown in the Supplementary Materials.

### 3.2. Dose–Response Analysis of Meat Intake and Mortality Risk

The dose–response analysis of red meat intake and all-cause mortality included 13 cohort studies with 28,875,891 person-years and 305,672 deaths [12,14,15,16,19,20,21,22,23,24,26,27,28], CVD mortality included 10 cohort studies with 18,663,795 person-years and 44,294 deaths [12,16,20,21,22,23,24,26,27,49], and cancer mortality included 10 cohort studies with 27,078,442 person-years and 106,331 deaths [12,15,16,20,21,22,23,24,27,49]. A linear dose–response relationship was observed between red meat intake and CVD mortality (*p*_nonlinearity_ < 0.001) and cancer mortality (*p*_nonlinearity_ < 0.001). For every additional 20 g of red meat consumed per day, the CVD and cancer mortalities increased by 4% (HR: 1.04; 95% CI 1.03–1.04) and 2% (HR: 1.02; 95% CI 1.02–1.03), respectively (Figure S4). There was a nonlinear dose–response relationship between red meat intake and all-cause mortality (*p*_nonlinearity_ < 0.05). (Figure S4).

The dose–response analysis of processed meat and all-cause mortality included 12 cohort studies with a total of 27,411,677 person-years and 295,519 deaths [12,14,15,16,19,20,21,22,23,24,27,28]; CVD mortality included 8 cohort studies with a total of 16,781,876 person-years and 38,733 deaths [12,16,20,21,22,23,24,27]; and cancer mortality included 9 cohort studies with a total of 25,369,466 person-years and 100,510 deaths [12,15,16,20,21,22,23,24,27]. Processed meat intake was associated with all-cause mortality, CVD mortality, and cancer mortality in a nonlinear dose–response relationship (all *p*_nonlinearity_ < 0.05). The risk of mortality positively is correlated with an increasing intake of processed meat products. Initially, the risk of mortality increased rapidly as the daily intake increased, but the trend tapered, and the increase in risk slowed as the intake exceeded 60–80 g/day (at which point, the risk of all-cause mortality increased by 26%, CVD mortality by 12%, and cancer mortality by 11%) (Figure S4).

Eight cohort studies were included in the dose–response analysis of mixed red and processed meat consumption and the risk of all-cause mortality [12,14,15,20,24,28,50,51], which included 15,763,369 person-years with 194,780 deaths. Five cohort studies for CVD mortality included 6,557,213 person-years with 15,994 deaths [12,20,24,50,51], and six cohort studies for cancer mortality included 15,144,987 person-years with 64,163 deaths [12,15,20,24,50,51]. A linear dose–response relationship between mixed meat consumption and CVD mortality was observed (*p*_nonlinearity_ = 0.9153). For every additional 40 g of mixed meat consumption per day, the risk of CVD mortality increased by 4% (HR: 1.04; 95% CI 1.03–1.05). A nonlinear dose–response relationship between mixed meat consumption and all-cause or cancer mortality was observed (*p*_nonlinearity_ < 0.05). The risk of mortality increases with the intake of mixed meat, but the trends became slower when the daily intakes exceed 240 g/day (at which point, the risk of all-cause mortality increased by 33% and cancer mortality by 23%) (Figure S4).

### 3.3. Dose–Response Analysis of Daily Steps, MSAs, and Mortality Risk

There were five cohort studies included in the dose–response analysis of daily steps and the risk of all-cause mortality [31,32,34,37,40] with 124,351 person-years and 956 deaths. We found a linear relationship between daily steps and all-cause mortality (*p*_nonlinearity_ = 0.4192), such that, for every additional 2000 steps per day, the risk of all-cause mortality decreased by 17% (HR: 0.83; 95% CI 0.79–0.86) (Figure S6). The dose–response of daily steps and mortality risk due to CVDs or cancer were not performed because of limited data.

Four cohorts were included in the dose–response analysis of MSAs and all-cause mortality with 2,004,873 person-years and 27,128 deaths [42,43,44,47]. Four studies examined the dose–response analysis between MSAs and CVD mortality with 2,018,472 person-years and 8031 deaths [42,43,44,47] and three studies for MSAs and cancer mortality with 1,916,240 person-years and 7875 deaths [42,44,47]. Significant nonlinear dose–responses were found between the length of time of MSAs per week and all-cause, CVD, and cancer mortalities (all *p*_nonlinearity_ < 0.05). The analyses indicated a J-shaped relationship between MSA time and the risk of mortality, with the risk being lowest at 30–40 min/week, but then, it increased sharply (Figure S5).

When MSA was 39.5 min/week, the risk of all-cause mortality was reduced by 20% (HR: 0.80; 95% CI 0.75–0.84), which was much higher than the effect for the comparisons of the highest versus lowest categories (0.91; 95% CI: 0.84–0.98). Conversely, when MSA was 30 min/week, the risk of CVD and cancer mortalities was reduced by 22% (HR: 0.78; 95% CI 0.70–0.87) and 13% (HR: 0.87; 95% CI 0.79–0.96), respectively.

### 3.4. Subgroup and Sensitivity Analyses

Subgroup analyses were performed based on the study region, methods of meat intake surveys, the number of participants, the meat intake unit, and the published year. The results showed that the source of heterogeneity in studies of red meat intake and cancer mortality may be caused by the number of participants (Table S5). Subgroup analyses on the relationship between MSAs and all-cause mortality were performed based on the study region, participation, follow-up years, and grouping criteria. The results showed that the source of heterogeneity may be the study region and the follow-up years (Table S5). Significant findings persisted across most subgroups, and there was little evidence of heterogeneity between most subgroups (Table S5). The results persisted in sensitivity analyses when excluding one study at a time from each analysis (Figures S9–S13).

### 3.5. GRADE Assessment

Because of statistical heterogeneity and publication bias, the certainty of the evidence was downgraded to very low for the associations of red meat intake and mortality; processed meat intake and the risk of mortality from all causes and CVDs; mixed red and processed meat intake and mortality; MSA and the risk of mortality from all-causes and cancer; and daily steps and all-cause mortality (Table S10).

### 3.6. Balanced Dose–Response Relationship between Meat Intake and Physical Activity

**Figure 3:**
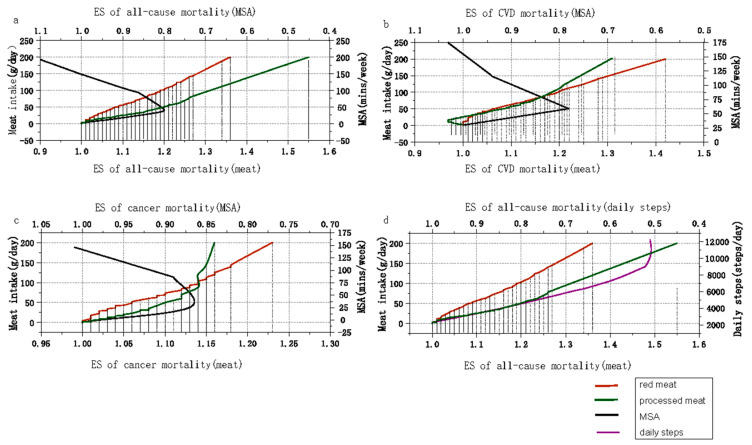
Balanced associations between red and processed meat intake and muscle-strengthening activity and daily steps. (**a**) Meat intake, MSA, and all-cause mortality; (**b**) meat intake, MSA, and CVD mortality; (**c**) meat intake, MSA, and cancer mortality; (**d**) meat intake, daily steps, and all-cause mortality.

As shown in Figure 3, the reduced risk (20%) from 39.5 min/week of MSA or 4100 steps/day was equivalent to the increased risk of all-cause mortality due to the intake of 103.4 g/day red meat or 50 g/day processed meat. The estimation of the allowable intake of red meat was higher for the same amount of risk than processed meat; the risk decreased further as the number of steps per day increased, but the risk was reversed when MSA exceeded the threshold (39.5 min/week) (Figure 3a,d, Table S9).

For the same risk of mortality, the consumption of red meat was initially greater than that of processed meat, whereas, as the risk of mortality increased further, the consumed amount of processed meat exceeded that of red meat. The reduced CVD mortality risk (22%) for 30 min/week of MSA was equivalent to the increased risk due to 105.40 g/day of red meat intake or 119.22 g/day of processed meat intake (Figure 3b, Table S9). Additionally, 30 min/week of MSA was equivalent to the 13% risk of cancer mortality associated with 88.35 g/day of red meat or 69.70 g/day of processed meat intake (Figure 3c, Table S9). Unlike the results for all-cause mortality risk, the tolerable intake of red meat was higher than that of processed meat when the increased risk of CVD (cancer) mortality was <16% (14%), and when this threshold was exceeded, processed meat had a greater tolerable intake.

## 4. Discussion

### 4.1. Findings of this Study

This study systematically investigated the trade-offs between red and processed meat intake and physical activity in long-term mortality outcomes. We compared the risk of mortality due to meat intake and physical activity separately and found that processed meat showed a higher all-cause mortality risk than red meat, while walking was more effective than MSA in reducing mortality risk but required longer activity.

### 4.2. Comparison with Other Studies and Mechanistic Insights

Numerous observational studies have shown that the high consumption of red meat and processed meat products is associated with an increased risk of mortality [52,53,54]. Our findings are consistent with those of recent meta-analysis studies that showed that red meat and processed meat consumption are associated with an increased risk of all-cause, CVD, and cancer mortality and that there is a positive dose–response relationship [53,54,55]. Interestingly, when comparing the dose responses, we further noted that red meat consumption was almost twice as high as processed meat consumption at the same risk of all-cause mortality. Processed meats are defined as products typically made from red meat that has been cured, salted, or smoked (e.g., ham or bacon) to improve the durability of the food product and/or to improve the color and taste and often contain large amounts of minced fatty tissue (e.g., sausages). Therefore, in contrast to red meat, at the same intake, processed meat may lead to an increased intake of saturated fats, cholesterol, salt, nitrite, polycyclic aromatic hydrocarbons, and heterocyclic amines [15,56]. All of these chemicals have been shown to cause chronic noncommunicable diseases, including cancer [57,58], CVDs [59,60], and diabetes [61]. This may explain why processed meat has a lower intake dose than red meat intake when the risk of all-cause mortality is the same.

By contrast, when we focused on CVDs and cancer, we found that, for the same risk of mortality, red meat consumption was initially greater than processed meat consumption, but with the increase in intake, the gap between the two gradually narrowed until the intake reached 84.86 g/day (CVDs) and 100 g/day (cancer), at which point, this was reversed. The biggest difference between processed and unprocessed meats is the number of preservatives, such as nitrites [62]. The risk of cancer in certain populations may be increased if the diet is high in nitrites. However, small doses of nitrite can lower blood pressure through vasodilatory properties, which may explain the differences we observed between red meat and processed meat intake in terms of cardiovascular disease and cancer mortality [63]. As is well known, the etiology of CVDs and cancer is complex, involving the interactions of genetic, environmental, and lifestyle factors. As a part of the diet, meat intake tends to be highly correlated with other diets and living habits. For example, high red and processed meat consumption, a hallmark of Western dietary patterns, typically involves the consumption of more than 100 g/day, together with the high consumption of sugar-sweetened beverages, refined grains, and nonmeat foods high in sodium, added sugar, and saturated fats [16,64]. This eating pattern is also associated with other unhealthy lifestyle behaviors, such as low physical activity, high alcohol intake, and tobacco use [1]. All of these may increase the risk of mortality from CVDs and cancer [65,66]. These findings emphasize the importance of distinguishing between meat types because processed meat appears to lead to higher mortality than unprocessed meat when total meat consumption cannot be limited to <100 g/day. However, an effort to limit the total amount of meat intake is the most effective method.

Physiological performance maintenance and health improvements generally result from regular physical activity, and most of these effects are highly predictable and dose-dependent and can be extended to various types of populations [7,67]. Walking, as an aerobic exercise, can improve body composition, lipids, insulin resistance, and inflammation. Epidemiological evidence suggests that 10 to 20 min of leisure aerobic exercise every day is sufficient to extend life expectancy [68], while high-level aerobic exercise (e.g., running) can provide additional survival benefits, which is 3–5 times the recommended minimum physical activity (75–150 min/week) at most, with an up to 10-fold-higher training volume generally considered to be safe and well-tolerated [69,70]. Recently, some studies aimed to determine the association between the number of steps per day and the risk of all-cause mortality [7,71]. Our dose–response meta-analysis also indicated a sharp linear inverse association, with an increase of 2000 steps per day associated with a 17% reduction in all-cause mortality, and the risk of mortality decreased proportionally with the increase in steps per day from 2000 to 12,000. Similar to the aforementioned biological mechanisms of walking, MSAs can increase muscle mass and strength, thereby improving glycemic control and insulin sensitivity. _ENREF_74 Moreover, MSAs are thought to reduce adiposity, such as visceral fat, and improve other mediating risk factors, including blood pressure, lipids, and lipoproteins [72]. However, for MSA, the dose–response curve for the risk of all-cause, CVD, and cancer mortalities in our research was J-shaped, suggesting that the greatest health benefits of MSA can be achieved when its duration is approximately 30–40 min/week. The meta-analysis by Giovannucci et al. also showed that, for CVD and all-cause mortality risks, the benefits of higher MSA levels (≥2.5 h/week) were less, or even detrimental, relative to lower MSA levels [8]. Although some studies have shown that high-intensity resistance training may have some adverse effects, such as increased arterial stiffness and myocardial hypertrophy [73], there is still insufficient evidence to explain why participation in MSA for a longer time has no additional benefits or why it may even reduce benefits. Training > 2.5 h (150 min) per week requires caution. Therefore, in terms of time efficiency, less time spent on MSA than walking reduced the risk of all-cause mortality by the same amount; therefore, it is recommended to conduct no more than 40 min/week of MSA to help reduce the risk of mortality. For walking, even a slight increase in steps per day was associated with a significantly reduced risk of all-cause mortality. However, health effect estimates for over 12,000 steps per day remain limited.

Epidemiologic and animal studies have clearly established that physical activity and diet play significant roles in healthy metabolism and disease prevention [74,75,76]. For example, a cohort study conducted by Ismael et al. examined the joint association between physical activity and Mediterranean diet scores and found that, among those who insisted on high physical activity and healthy diet scores, the risk of all-cause mortality was reduced the most [77]. A few experimental studies involving human subjects have indicated that high-intensity exercise frequently counteracts the negative metabolic consequences of overeating, including insulinemic responses, cardiometabolic profiles, and gene expression in adipose tissue [75,76]. In a short-term experimental study on mice conducted by Gioscia-Ryan et al., a Western diet worsened age-related endothelial dysfunction, vascular oxidative stress, and aortic inflammation, while exercise lessened or even reversed all these effects [74]. The fundamental metabolic pathways that food and exercise might use to impair and improve health, respectively, are substantially the same [67]. However, the mechanisms by which diet and physical activity interact and how to achieve the optimal combination of the two for benefit remain elusive. In our study, we partially visualized the balance of mortality risk between physical activity and red or processed meat intake using dose–response relationship graphs. For all-cause mortality, consuming 103.4 g of red meat or 50 g of processed meat per day increased the risk by 20%, and these risks were equivalent to the risk reduction associated with walking and MSA requiring 4100 steps/day and 39.5 min/week, respectively. These findings imply that, when one consumes red or processed meat in one’s diet, one may be able to mitigate the increased mortality risk associated with red and processed meat intake by engaging in a certain amount of physical activity, from simple walking to time-saving MSA. Overall, the main implication of our results is to help people adjust their physical activity patterns and durations according to their daily intake of red and processed meat by referring to the balanced scale we provided and thereby reduce the possible increased mortality due to the adverse physiological outcomes of the long-term consumption of excessive red and processed meat.

### 4.3. Strengths and Weaknesses of this Study

The most significant strength of this meta-analysis is that, for the first time, risk factors for mortality were jointly analyzed with protective factors to infer a balanced association between red and processed meat intake and daily step count and MSA from a dose–response meta-analysis. Since only the effect sizes with the most complete adjustment for confounding factors were included, the stability of the results was ensured to the greatest extent. In addition, a study [78] based on the UK Biobank population examined independent and interactive associations between physical activity and diet and all-cause and CVD, and no additive or multiplicative interactions between physical activity categories and dietary quality was found. Therefore, this means that the equilibrium relationship we inferred should exist even in the same population in the real world, rather than just a model-based relational assumption. Second, we determined the reference intake dose of red meat versus processed meat at the same mortality risk and the MSA time threshold for the most efficient reduction in mortality risk based on the dose–response correlation curves so as to recommend more appropriate protein sources and exercise modalities. Third, all included studies were prospective, and recall bias and selection bias were negligible. Finally, the large number of participants and mortalities allowed for a more accurate quantitative assessment of the association between meat intake, MSA, daily steps, and mortality, making it more reliable than any single study.

This study has some limitations. First, when estimating meat intake, measurement error is inevitable. Second, there was obvious heterogeneity between the included studies, which may have resulted from differences in follow-ups, exposure assessment methods, frequency of dietary assessments, and adjustments of confounding factors. Heterogeneity and publication bias both reduce the certainty of meta-analysis evidence, thus affecting the accuracy of the balance scale estimation. Third, we quantified the balanced relationship between meat intake and physical activity based on the point estimation of dose–response curves in the meta-analysis, which still requires conservative and strict interpretation. Fourth, a high level of physical activity cannot completely compensate for the adverse impact of an unhealthy diet on the physiology and mortality risk [78]; what we proposed is purely a preventive balance reference. Finally, in our study, the search deadline was December 2021, but we recently searched and found no other studies of high quality from a different database.

## 5. Conclusions

We revealed balanced associations for the effect between dietary meat intake and physical activity on mortality risk in the present analysis. Our findings suggested that processed meat may represent a higher risk of mortality than red meat. Unlike walking, a longer time spent on MSA did not correspond to a stronger protective effect after the risk threshold was exceeded. We provided a balanced scale of mortality risk associated with red and processed meat intake and that of daily steps and MSA duration, which may lead to the design of more effective and efficient interventions and policies.

## Figures and Tables

**Table 1:** Characteristics of included studies for association between meat intake and all-cause mortality.

Author, Year of Publication, Country	Age *	No. of Participants	Follow-Up (Years) †	No. of Deaths	Exposure	Exposure Assessment	Comparison of Meat Intake	Effect Size (95% CI) §	Adjustment
Zhong, 2019,	53.7	29,682	19	8875	Red meat	FFQ	2 vs. 0 servings/week	HR 1.03 (1.01–1.05)	1,2,3,4,5,6,7,8,9,10,11,
US					Processed		2 vs. 0 servings/week	HR 1.03 (1.02–1.05)	12,13,14,15,16,17,18
Piet A, 2019,	55–69	M 58,279	10	5797	Red meat	FFQ	140.4 vs. 41.3 g/day	HR 1.02 (0.86–1.2)	1,2,4,5,6,9,11,14,19,20,
The Netherlands		W 62,573		3026	Processed		30.8 vs. 0 g/day	HR 1.21 (1.02–1.44)	21,22,23,24,25,26,27
Zheng, 2019,	M 30–55	27,916	409,073 **	5593	Red meat	FFQ	>0.5/change of <0.15 serving/day	HR 1.10 (1.04–1.17)	1,3,6,8,9,21,22,23,24,28
US	W 40–75	53,553	804,685 **	8426	Processed		>0.5/change of <0.15 serving/day	HR 1.13 (1.04–1.23)	29,30,31,32,33,34,35,36
Alshahrani,	>25	72,149	11.8	7961	Red meat	FFQ	41.7 vs. 4 g/day	HR 1.17 (1.05–1.32)	1,2,3,4,5,6,9,18,21,22,
2019, US					Processed		9.4 vs. 0.7 g/day	HR 1.16 (1.04–1.29)	24,32,33,34,35,36,37,38
and Canada					Combined		42.8 vs. 1.4 g/day	HR 1.25 (1.12–1.40)	39,40,41,42,43,44,45,4647,48
Mejborn, 2020,	15–75	9848	NR	640	Red meat	7-day pre-coded	>97 vs. <41 g/day	HR 0.86 (0.67–1.12)	2,3,4,5,6,8,9,24
Denmark					Processed	food diary	>58 vs. <19 g/day	HR 1.02 (0.82–1.26)	
Argyridou, 2019, UK	40–69	419,075	7	15,058	Combined	FFQ	7.0 vs. 1.5 servings/week	HR 1.252 (1.172–1.338)	1,2,3,4,6,8,9,11,14,24, 47,48,49,50,51,52
Dominguez,	>45	18,540	9.5	255	Red meat	136-item	>7 vs. <3 servings/week	HR 1.86 (1.19–2.93)	2,4,5,6,9,24,25,53,56,
2017,					Processed	FFQ	>7 vs. <3 servings/week	HR 1.57 (0.76–3.24)	74,82,83,84
Spain					Combined		>7 vs. <3 servings/week	HR 1.31 (0.75–2.30)	
Etemadi,	50–71	M 316,505	15.6	84,848	Red meat	124-item	50.3 vs. 6.9 g/1000 kcal	HR 1.20 (1.17–1.22)	1,2,3,4,5,6,8,9,11,14,24,
2017, US		W 220,464		43,676	Processed	FFQ	17.2 vs. 2.3 g/1000 kcal	HR 1.15 (1.13–1.17)	29,30,37,54,55,56,57,58
Sheehy,	38	W 56,314	22	5054	Red meat	FFQ	1.0 vs. 0.01 serving/day	HR 1.47 (1.33–1.62)	4,5,6,8,21,24,28,30,60,
2020, US					Processed		1.2 vs. 0.01 serving/day	HR 1.40 (1.28–1.55)	61,62
Saito,	45–74	M 40,072	14	6266	Red meat	FFQ	92.9 vs. 14.3 g/d	HR 1.13 (1.02–1.26)	1,5,6,9,11,14,15,16,18,
2020, Japan					Processed		8.4 vs. 1.3 g/d	HR 0.98 (0.91–1.07)	21,22,24,47,63,64,65
		W 47,435		3620	Red meat		90.3 vs. 13.6 g/d	HR 1.08 (0.95–1.24)	
					Processed		11.7 vs. 2.1 g/d	HR 1.05 (0.95–1.17)	
Rohrmann,	35–69	448,568	12.7	26,344	Red meat	FFQ	160+ vs. 10–19.9 g/d	HR 1.10 (0.98–1.24)	1,2,4,5,6,7,8,9,24,31,59
2013, Europe					Processed		160+ vs. 10–19.9 g/d	HR 1.43 (1.24–1.64)	
Sinha,	50–71	500,000	10	M 47,976	Red meat	124-item	68.1 vs. 9.3 g/1000 kcal	HR 1.31 (1.27–1.35)	4,6,8,9,11,14,19,24,26
2009, US					Processed	FFQ	19.4 vs. 5.1 g/1000 kcal	HR 1.16 (1.12–1.19)	30,37,66
				W 23,276	Red meat		65.9 vs. 9.1 g/1000 kcal	HR 1.36 (1.30–1.43)	
					Processed		16.0 vs. 3.8 g/1000 kcal	HR 1.25 (1.20–1.31)	
Pan,	NA	M 37,698	22	M 8926	Red meat	FFQ	2.36 vs. 0.22 servings/day	HR 1.29 (1.20–1.38)	1,3,5,6,8,9,11,14,21,22,
2012, US					Processed		2.36 vs. 0.22 servings/day	HR 1.27 (1.19–1.36)	24,28,29,30,34,35,36,43
					Combined		2.36 vs. 0.22 servings/day	HR 1.37 (1.27–1.47)	
		W 83,644	28	W 15,000	Red meat		3.1 vs. 0.53 servings/day	HR 1.19 (1.13–1.25)	
					Processed		3.1 vs. 0.53 servings/day	HR 1.20 (1.14–1.27)	
					Combined		3.1 vs. 0.53 servings/day	HR 1.24 (1.17–1.30)	
Takata,	40–74	M 61,483	334,281 **	2733	Red meat	FFQ	114.9 vs. 20.0 g/day	HR 1.18 (1.02–1.35)	1,4,5,6,7,8,9,11,14,47,
2013, China		W 74,941	803,265 **	4210	Red meat		94.8 vs. 15.0 g/day	HR 0.92 (0.82–1.03)	48,59,67,68,69
Bellavia, 2016,	45–83	M 40,089	16	10,423	Red meat	FFQ	140 vs. 31 g/day	HR 1.21 (1.13–1.29)	2,4,5,7,8,9,22,24,47
Sweden		W 34,556		7486					
Kappeler, 2013, US	>18	17,611	22	M 1908	Red meat Processed	FFQ	45+ vs. 0–6 times/week 45+ vs. 0–6 times/week	HR 1.24 (0.76–2.02) HR 1.06 (0.75–1.50)	1,2,3,6,8,9,11,14,21,22,24,26,29,32,35,36,37,61
				W 1775	Red meat		45+ vs. 0–6 times/week	HR 1.49 (0.76–2.94)	73,74
					Processed		45+ vs. 0–6 times/week	HR 1.16 (0.86–1.55)	
Lee, 2013,	17–92	M 112,310	6.6–15.5	23,515	Red meat	FFQ	Q4/Q1	HR 0.93 (0.84–1.02)	1,4,5,6,9,11,14,24,76
Asian		W 184,411		16,699	Red meat		Q4/Q1	HR 0.93 (0.86–1.00)	
Whiteman,	35–64	10,522	9	514	Red meat	FFQ	4–7 vs. <1 day week-1	HR 0.71 (0.55–0.92)	1,2,6
1999, UK					Processed		4–7 vs. <1 day week-1	HR 1.05 (0.62–1.76)	
Farvid, 2016, Iran	51.6	42,403	11	3291	Red meat		0.43 vs. 0.02 serving/day	HR 1.04 (0.93–1.17)	1,2,3,4,5,6,8,9,24,30,37,51,70,76
Iqbal, 2021,	35–70	134,297	9.5	7789	Red meat	FFQ	≥250/<50 g/week	HR 0.93 (0.85, 1.02)	1,2,4,5,6,8,11,12,14,
21 countries					Processed		≥250/<50 g/week	HR 1.51 (1.08, 2.10)	41,22,78,79,80
Sun, 2021,	50–79	102,521	18.1	25,976	Red meat	FFQ	3.2/0.3 oz equivalent/d	HR 1.05 (0.99–1.10)	1,3,4,5,6,8,9,11,14,17
US					Processed		1.0/0.01 oz equivalent/d	HR 1.06 (1.01–1.10)	22,41,43,67,71,74,81
					Combined		3.9/0.4 oz equivalent/d	HR 1.10 (1.05–1.15)	

FFQ = food frequency questionnaire; HR = hazard ratio; M = men CI = confidence interval; NR = not reported; W = women. * Presented as mean or range. ** Person-years. † Number of years that individuals were followed up in the prospective cohort studies. § These effect sizes are for comparison of the highest and the lowest categories. Adjustments: age (1), sex (2), race/ethnicity (3), educational level (4), total energy (5), smoking status (6), smoking pack-years (7), physical activity (8), alcohol intake (9), hormone therapy (10), fruits (11), legumes (12), potatoes (13), vegetables (14), low-fat dairy products (15), high-fat dairy products (16), sugar-sweetened beverages (17), eggs (18), number of cigarettes smoked per day (19), years of smoking (20), history of physician-diagnosed hypertension (21), history of physician-diagnosed diabetes (22), body height (23), BMI (24), non-occupational physical activity (25), use of nutritional supplements (26), in women postmenopausal HRT (27), family history of myocardial infarction (28), family history of diabetes (29), family history of cancer (30), weight (31), aspirin use (32), multivitamin use (33), menopausal status (34), postmenopausal hormone therapy use for women (35), physician-diagnosed hypercholesterolemia (36), marital status (37), exercise (38), sleep (39), the use of statin (40), the use of blood pressure medications (41), cruciferous vegetables (42), whole grain (43), nuts (44), seeds (45), total dairy (46),fish (47), unprocessed poultry (48), Townsend score (49), employment (50), number of medications (51), salt added to food (52), time TV viewing (53), quintiles of a composite deprivation index (54), perceived health at baseline (55), history of heart disease (56), history of stroke (57), usual activity throughout the day (58), total meat intake (59), geographic region (60), neighborhood SES (socioeconomic status) (61), Alternative Healthy Eating Index without red meat (62), quartile of metabolic equivalent task-hours/d (63), sodium (64), total fat (65), time since quitting for former smokers (66), income (67), occupation (68), comorbidity index (69), systolic blood pressure (70), proteinuria (71), overall health (72), use of ibuprofen (73), family history of hypercholesterolemia (74), history of gallstones (75), residency (76), opium use (77), wealth score (78), location (79), starchy foods (80), unopposed estrogen use (81), year of entering the cohort (82), history of depression (83), following special diets at baseline (84).

**Table 2:** Characteristics of included studies for association between muscle-strengthening activity and all-cause mortality.

Author, Year of Publication, Country	Age *	Sample size	Follow-Up (Years) †	No. of Cases	Exposure	Exposure Assessment	Comparison of Meat Intake	Effect Size (95% CI) §	Adjustment
Zhao, 2020,	≥18	479,856	8.75	59,819	MSA	Self-reported	<2 times/week	HR 1	1,2,3,4,5,6,7,8,9
US							≥2 times/week	HR 0.89 (0.85–0.94)	
Stamatakis,	≥19	72,459	9.2	5763	Strength-	Questionnaire	None	HR 1	1,2,4,6,7,8,11,12
2017, UK					promoting exercise		Any	HR 0.77 (0.69–0.87)	
Kamada, 2017,	62.2	28,879	12	3055	Strength	Questionnaire	0	RR 1	2,3,4,6,7,8,13,14,15,16,
US	(Mean)				training		≥150 min/week	RR 1.10 (0.77–1.56)	17,18,19,20,21,22,23,24,25,26,27,28
LIU, 2018, US	18–89	12,591	10	276	Resistance	Questionnaire	0	HR 1	1,2,6,7,8,24,27,29,30
					exercise		≥120 min/week	HR 1.03 (0.59–1.80)	
Patel,	70.2	72,462	13	17,750	MSA	Questionnaire	0	HR 1	1,2,4,5,6,7,8,31,32,33,
2020, US							≥2 h/week	HR 1.01 (0.93–1.09)	34,35,36,37
Porter, 2020,	46.3	17,938	11.9	3799	Weightlifting	Questionnaire	No	HR 1	1,2,3,4,6,7,8,38
US	(Mean)						Yes	HR 0.89 (0.67–1.17)	
Hsu, 2017,	≥70	1705	7	519	Muscle-	Questionnaire	No	HR 1	2,3,4,6,7,8,26,27,36,50,
Australia					strengthening exercise		Yes	HR 0.72 (0.49–1.06)	51,52,53
Sheehan,	18–84	26,727	17	4955	Weightlifting	Questionnaire	No	HR 1	1,2,3,4,5,6,7,8,50,51,54,
2020, US							Yes	HR 0.95 (0.85–1.07)	55,56,57

MSA = muscle-strengthening activity; HR = hazard ratio; RR = risk ratio; CI = confidence interval; CVD = cardiovascular disease; NR = not reported; * Presented as mean or range. † The number of years that individuals were followed up in the prospective cohort studies. § These effect sizes are for comparison of the highest and the lowest categories. Adjustments: sex (1), age (2), race/ethnicity (3), education (4), marital status (5), body mass index (6), smoking status (7), alcohol intake (8), chronic conditions (9), long-standing illness (10), psychological distress (11), weekly physical activity volume excluding the volume of strength-promoting activity (12), trial randomization (13), postmenopausal status (14), hormone use (15), parental history of myocardial infarction or cancer (16), energy intake (17), saturated fat intake (18), fiber intake (19), fruit and vegetable intake (20), physical examination for screening (21), time per week spent in aerobic activity (22), MVPA (for strength training and vice versa) (23), incidence of hypertension (24), high cholesterol (25), cardiovascular diseases (26), diabetes mellitus (27), cancer before and during follow-up (28), parental history of CVD (29), hypercholesterolemia (30), survey type (31), self-reported overall health (32), work status (33), TV sitting time (34), aspirin use (35), comorbidity score (36), aerobic moderate-to-vigorous physical activity (37), household (38), use of an ambulatory device (39), total cholesterol level (40), statin medication use (41), measured mean arterial blood pressure (42), following physician-diagnosed conditions (43), arthritis (44), stroke (45), serum C-reactive protein (46), congestive heart failure (47), coronary artery disease (48), emphysema (49), self-rated health (50), ADL disability (51), depression (52), PASE score (53), income (54), nativity status (55), census region of residence (56), home ownership (57), eGFR (58), need special equipment to walk (59).

**Table 3:** Characteristics of included studies for association between daily steps and all-cause mortality in adults aged 45 or older.

Author, Country	Age *	Sample Size	Follow-Up (Years) †	No. of Cases	Exposure	Exposure Assessment	Number of Steps per Day	Effect Size (95% CI) §	Adjustment
Lee, 2019, US	≥45	16,741	4.3	504	Steps	ActiGraph	2718	HR 1	1,2,3,4,5,6,7,8,9,10,11,12,
						GT3X+ accelerometers	4363	HR 0.59 (0.47–0.75)	13,14
							5905	HR 0.54 (0.41–0.72)	
							8442	HR 0.42 (0.3–0.6)	
Hansen, 2020,	57	2183	9.1	119	Steps	ActiGraph, LLC,	4651	HR 1	2,3,4,15,16,21,22,23
Norway						Pensacola, FL	6862	HR 0.52 (0.29–0.93)	
							8670	HR 0.5 (0.27–0.94)	
							11,467	HR 0.43 (0.21–0.88)	
Maurice, 2020,	56.8	4840	10.1	1165	Steps	ActiGraph 7164	4000	HR 1	1,3,4,12.15.16.22,24,26,27,
US							8000	HR 0.49 (0.44–0.55)	28,29,30
							12,000	HR 0.35 (0.28–0.45)	
Jefferis, 2017, UK	78.4	1181	5	194	Steps	ActiGraph GT3x	1895	HR 1	1,2,3,4,22,25,33,34,35,
							3646	HR 0.63 (0.43–1.54)	36,37
							5302	HR 0.59 (0.39–0.9)	
							8401	HR 0.31 (0.17–0.57)	
Yamamoto, 2018,	71	419	9.8	76	Steps	spring-levered	3394	HR 1	3,4,15,22,23
Japan						pedometer (EC-100S,	5310	HR 0.81 (0.43–1.54)	
						YAMASA, Tokyo,	6924	HR 1.26 (0.7–2.26)	
						Japan)	10,241	HR 0.46 (0.22–0.96)	
Oftedal, 2019, Australia	65.4	1697	9.6	NR	Steps	DigiwalkerSW-200 pedometer	Per 1000 steps per day	HR 0.93 (0.88–0.98)	1,3,15,38
Dwyer, 2015,	58.8	2576	10	219	Steps	Omron HJ-003	0–5550	HR 1	1,3,4,15,22,39
Australia						Omron HJ-102	5551–8000	HR 0.43 (0.30–0.62)	
							8001–10,000	HR 0.25 (0.16–0.38)	
							10,001–13,500	HR 0.24 (0.15–0.37)	
							13,501–39,164	HR 0.10 (0.05–0.18)	
Manas, 2021, Spain	78.8	768	5.7	89	Steps	ActiTrainer ActiGraphwGT3X-BT; ActiGraph, Pensacola,	per additional 1000 steps	HR 0.87 (0.81–0.95)	1,2,15,16,22,38,40,41
Paluch, 2021, US	45.2	2110	10.8	72	Steps	ActiGraph 7164	5837	HR 1	1,2,3,4,12,13,15,16,22,23,
							8502	HR 0.28 (0.15–0.54)	25,26,42,43,44
							11,815	HR 0.45 (0.25–0.81)	
Klenk, 2016, US	75.6	1271	4	100	Walking duration	activPAL, PAL Technologies Ltd., Glasgow, UK	128.4–290.5 vs. 3.7–76.1 min/day	HR 0.39 (0.19–0.78)	1,3,4,13,14,15,16,22,26,31,44
Pate, 2017, US	69.8	139,255	13	43,621	Walking duration	assessed by asking	>6 h/week vs. <2 h/week	HR 0.78 (0.75–0.81)	4,7,8,25,26,39,40,45,46,47

* Presented as mean or range. † The number of years that individuals were followed up in the prospective cohort studies. § These effect sizes are for comparison of the highest and the lowest categories. Age (1), wear time (2), smoking status (3), alcohol use (4), intake of saturated fat (5), intake of saturated fiber (6), intake of saturated fruits (7), intake of saturated vegetables (8), hormone therapy (9), parental history of myocardial infarction (10), family history of cancer (11), general health (12), history of cardiovascular disease (13), history of cancer and cancer screening (14), gender (15), educational attainment (16), IMD (17), weight status (18), GP Management System (19), number of self-reported chronic illnesses at baseline (20),VPA (21), body mass index (22), number of medical conditions (23), diet quality (24), race/ethnicity (25), diabetes (26), stroke (27), coronary heart disease (28), heart failure (29), cancer (30), chronic bronchitis (31), emphysema (32), mobility limitation (33), season of wear (34), social class (35), sleep time (36), living alone (37), household income (38), total energy intake from all sources (kJ) (39), marital status (40), comorbidities (41), study center (42), systolic blood pressure (43), hyperlipidemia (44), red/processed meat intake (45), occupational status (46), leisure-time sitting (47).

## Data Availability

No new data were created or analyzed in this study. Data sharing is not applicable to this article.

## Supplementary Materials

The following supporting information can be downloaded at https://www.mdpi.com/article/10.3390/nu15153373/s1: Table S1: Medical subject headings (MeSH) and non-MeSH keywords used to search relevant publications. Table S2: Characteristics of included studies for association between meat intake and mortality from cardiovascular disease and cancer. Table S3: Characteristics of included studies for association between muscle-strengthening activity and mortality from cardiovascular disease and cancer. Table S4: Results of risk of bias assessment based on the Newcastle-Ottawa scale. Table S5: Association of meat intake, daily steps and muscle-strengthening activity for different subgroups. Table S6: The balanced relationship between meat intake and physical activity (all-cause mortality). Table S7: The balanced relationship between meat intake and physical activity (cardiovascular disease mortality). Table S8: The balanced relationship between meat intake and physical activity (cancer mortality). Table S9: The balanced relationship between meat intake and physical activity. Table S10: The pooled estimates of red and processed meat, physical activity and all-cause, cardiovascular disease and cancer mortality. Table S11: PRISMA Checklist of the systematic review and meta-analysis. Table S12: PRISMA Abstracts Checklist of the systematic review and meta-analysis. Table S13: Number of studies from different databases. Figure S1: Forest plots of meta-analyses of red and processed meat intake and mortality risk. Figure S2: Forest plots of meta-analyses of mixed intake of red and processed meat and mortality risk. Figure S3: Forest plots of meta-analyses of muscle-strengthening activity (MSA) and mortality outcomes. Figure S4: Linear and non-linear dose-response relationships between red and processed meat intake and mortality risk. Figure S5: Non-linear dose-response relationships between MSA and mortality risk. Figure S6: Linear dose-response relationships between daily steps all-cause mortality. Figure S7: Balanced associations between mixed intake of red and processed meat and muscle-strengthening activity. Figure S8: Balanced associations between mixed intake red and processed meat, daily steps and all-cause mortality. Figure S9: Sensitivity analysis of mixed red and processed intake and mortality outcomes. Figure S10: Sensitivity analysis of red meat intake and mortality outcomes. Figure S11: Sensitivity analysis of processed intake and mortality outcomes. Figure S12: Sensitivity analysis of muscle-strengthening activity (MSA) and mortality outcomes. Figure S13: Sensitivity analysis of daily steps and all-cause mortality. Figure S14: Funnel plot of included studies in the meta-analysis of the intake of mixed red and processed meat and mortality from all-cause, cardiovascular disease and cancer. Figure S15: Funnel plot of included studies in the meta-analysis of the intake of red meat and mortality from all-cause, cardiovascular disease, and cancer. Figure S16: Funnel plot of included studies in the meta-analysis of the intake of processed meat and mortality from all-cause, cardiovascular disease, and cancer. Figure S17: Funnel plot of included studies in the meta-analysis of daily steps and all-cause mortality. References [79,80,81,82,83,84,85,86] are mentioned in Supplementary Materials.
