# Supplementary material for: How to Keep the Balance between Red and Processed Meat Intake and Physical Activity Regarding Mortality: A Dose-Response Meta-Analysis
Source: Nutrients. 2023 Jul 29;15(15):3373. doi: 10.3390/nu15153373 (PMC10421417; doi:10.3390/nu15153373)
Supplement: Supplementary file 1 [file nutrients-15-03373-s001.zip › Supplementary Materials .pdf]

Supplementary Materials

How to keep the balance between red or processed meat intake and physical activity on mortality: a dose-response meta-analysis

Content 3

Supplementary Results **5**

Characteristics of included studies 5

Meta-analysis on red and processed meat intake and mortality risk 5

Meta-analysis on muscle-strengthening activities and mortality risk 6

Meta-analysis on daily steps and all-cause mortality 7

Supplementary References 7

Supplementary Tables 11

Supplementary Table S1: Medical subject headings (MeSH) and non-MeSH keywords used to search relevant publications 11

Supplementary Table S2: Characteristics of included studies for association between meat intake and mortality from cardiovascular disease and cancer 12

Supplementary Table S3: Characteristics of included studies for association between muscle-strengthening activity and mortality from cardiovascular disease and cancer 20

Supplementary Table S4: Results of risk of bias assessment based on the Newcastle-Ottawa scale 24

Supplementary Table S5: Association of meat intake, daily steps and muscle-strengthening activity for different subgroups 28

Supplementary Table S6: The balanced relationship between meat intake and physical activity (all-cause mortality) 33

Supplementary Table S7: The balanced relationship between meat intake and physical activity (cardiovascular disease mortality) 34

Supplementary Table S8: The balanced relationship between meat intake and physical activity (cancer mortality) 35

Supplementary Table S9: The balanced relationship between meat intake and physical activity 36

Supplementary Table S10: The pooled estimates of red and processed meat, physical activity and all-cause, cardiovascular disease and cancer mortality 38

Supplementary Table S11: PRISMA Checklist of the systematic review and meta-analysis 40

Supplementary Table S12: PRISMA Abstracts Checklist of the systematic review and meta-analysis 43

Supplementary Table S13: Number of studies from different databases......................... 44

Supplementary Figures 46

Supplementary Figure S1: Forest plots of meta-analyses of red and processed meat intake and mortality risk..46

Supplementary Figure S2: Forest plots of meta-analyses of mixed intake of red and processed meat and mortality risk 47

Supplementary Figure S3: Forest plots of meta-analyses of muscle-strengthening activity (MSA) and mortality outcomes 48

Supplementary Figure S4: Linear and non-linear dose-response relationships between red and processed meat intake and mortality risk...49

Supplementary Figure S5: Non-linear dose-response relationships between MSA and mortality risk 50

Supplementary Figure S6: Linear dose-response relationships between daily steps all-cause mortality 51

Supplementary Figure S7: Balanced associations between mixed intake of red and processed meat and muscle-strengthening activity 52

Supplementary Figure S8: Balanced associations between mixed intake red and processed meat, daily steps and all-cause mortality.......................................53

Supplementary Figure S9: Sensitivity analysis of mixed red and processed intake and mortality outcomes...54

Supplementary Figure S10: Sensitivity analysis of red meat intake and mortality outcomes...55

Supplementary Figure S11: Sensitivity analysis of processed intake and mortality outcomes 56

Supplementary Figure S12: Sensitivity analysis of muscle-strengthening activity (MSA) and mortality outcomes 57

Supplementary Figure S13: Sensitivity analysis of daily steps and all-cause mortality .58

Supplementary Figure S14: Funnel plot of included studies in the meta-analysis of the intake of mixed red and processed meat and mortality from all-cause, cardiovascular disease and cancer 59

Supplementary Figure S15: Funnel plot of included studies in the meta-analysis of the intake of red meat and mortality from all-cause, cardiovascular disease, and cancer 60

Supplementary Figure S16: Funnel plot of included studies in the meta-analysis of the intake of processed meat and mortality from all-cause, cardiovascular disease, and cancer...61

Supplementary Figure S17: Funnel plot of included studies in the meta-analysis of daily steps and all-cause mortality 61

Supplementary Results

Characteristics of included studies

All studies were published between 1999 and 2021 and had a prospective cohort design. Most studies (26/57) were conducted in the USA. Other studies came from countries and regions including the Netherland (1), Denmark (1), China (1), the UK (6), Australia (3), Norway (1), Iran (1), Japan (3), Spain (2), Sweden (1), Asia (1), European (1) and 21 countries (1). The maximum follow-up is 30 years and participants' age ranged from 18 to 92. All studies except for eight[1-8] adjusted for age. Most cohorts controlled for some conventional risk factors, including sex (in n=28 studies), smoking (n=38), and alcohol consumption (n=35). Others also adjusted for race/ethnicity (n=19), education (n=29), body mass index (n=33), and marital status (n=11). To examine meat intake, 26 studies used a food frequency questionnaire and one publication used dietary records. To measure step counts per day, seven studies used an accelerometer and three studies used a pedometer, one study used a questionnaire to record walking time. Eight studies used questionnaire to measure MSAs and one study used self-reporting methods.

Meta-analysis on red and processed meat intake and mortality risk

Of 27 studies included 5,893,740 participants and 435,880 deaths on the intake of red meat and processed meat and mortality[1-5, 9-30] risk, 21 studies with 3,317,459 participants and 426,936 deaths presented sufficient data for the comparison of the highest versus lowest categories of red and processed meat intake and all-cause mortality[1-5, 9-16, 18, 20, 21, 25-27, 29, 30], 16 of CVD mortality included 2,616,909 participants and 250,719 deaths[2-4, 9, 10, 13-16, 18, 20-22, 25-27], and 20 of cancer mortality included 4,898,161 participants and 382,793 deaths[2-4, 9-16, 18-21, 23-27].

The association between consumption of red meat and CVD mortality was examined in 13 papers, the summary effect size (pooled HR) comparing the highest and lowest intakes was (1.14; 95% CI 1.03 to 1.26), with significant heterogeneity (I^2=85% P < 0.01) (Fig. S1) [2, 4, 9, 14-16, 18, 20-22, 25-27]. For red meat intake and cancer mortality, an analogous result (HR 1.07; 95% CI 1.01 to 1.13) (Fig. S1) was obtained based on 16 publications with significant heterogeneity (I^2=77% P < 0.01) [2, 4, 9, 11, 12, 14-16, 18-21, 24-27]. No evidence of publication bias was detected (Egger's test, P=0.208 for CVD

mortality, *P*= 0.167 for cancer mortality; Begg's test, *P*=0.596 for CVD mortality, *P*=0.695 for cancer mortality) (Table S10).

Of 10 papers[2, 4, 9, 14, 15, 18, 20, 21, 25, 27], significant associations between consumption of processed meat and CVD mortality were found (HR1.21; 95% CI 1.08 to 1.35) (Fig. S1) with significant heterogeneity (I^2=83% P < 0.01).Ae similar result was obtained in cancer mortality, which was examined in 15 articles (HR1.11; 95% CI 1.09 to 1.14) (Fig. S1) [2, 4, 9, 11, 12, 14, 15, 17-21, 23, 25, 27]. Moreover, no significant heterogeneity among the studies was found (I^2=2% *P*=0.43). No evidence of publication bias was detected (Egger's test, *P*=0.428 for CVD mortality, *P*= 0.759 for cancer mortality; Begg's test, *P*=0.760 for CVD mortality, *P*=0.675 for cancer mortality) (Table S10).

The association between consumption of mixed red and processed meat and all-cause mortality was examined in ten papers[2, 3, 5, 9, 10, 12, 13, 18, 25, 29], the summary effect size was 1.22 (95% CI 1.15 to 1.30) (Fig. S2). Significant heterogeneity was seen between studies (I^2=86%, *P*<0.01). Mixed consumption of red and processed meat, which was examined in eight studies was associated with increased mortality of CVD (HR 1.30; 95% CI 1.20 to 1.41) (Fig. S2)[2, 3, 9, 10, 13, 18, 25, 28], with moderate heterogeneity among the studies (I^2=67% P < 0.01). Eight studies examined the association between mixed consumption of red and processed meat and cancer mortality (HR 1.15; 95% CI 1.12 to 1.19) (Fig. S2) with low heterogeneity (I^2=46% *P*=0.08) [2, 3, 9, 10, 12, 13, 18, 25]. No evidence of publication bias was detected (Egger's test, *P*=0.85 for all-cause mortality, *P*= 0.485 for CVD mortality; Begg's test, *P*=0.876 for all-cause mortality, *P*=0.917 for CVD mortality) and little evidence of publication bias was observed in the cancer mortality analysis (*P*=0.159 in Egger's test, *P*=0.037 in Begg's test) (Table S10).

Meta-analysis on muscle-strengthening activities and mortality risk

Among the 9 studies included a total of 1,023,245 participants and 103,211 deaths investigating MSAs and mortality[31-39], 8 studies with 712,617 participants and 95,936 deaths provided enough data for comparison of the highest and lowest categories of MSAs with mortality from all causes[31-36, 38, 39]; 7 studies with 685,890 participants and 90,981 deaths for CVD mortality[31-35, 38, 39]; and 7 studies with 983,927 participants and 97,980 deaths for cancer mortality[31, 32, 34, 35, 37-39].

In studies on the relationship between MSA and CVD mortality, MSAs were associated with a 12% lower risk of CVD mortality (HR 0.88; 95% CI 0.81 to 0.95) (Fig. S3) with moderate heterogeneity among the studies (I^2=43% *P*=0.11). Similar results were also observed in the relationship between MSA and cancer mortality (HR 0.85 (95% CI 0.76 to 0.94) (Fig. S3), evidence of moderate heterogeneity was found between studies (I^2=54%, *P*=0.04). No evidence of publication bias was detected (Egger's test, *P*=0.380 for CVD mortality, *P*= 0.763 for cancer mortality; Begg's test, *P*=1 for CVD mortality, *P*=1 for cancer mortality) (Table S11).

Meta-analysis on daily steps and all-cause mortality

The data of 11 studies included a total of 173,041 participants and 46,159 deaths[6-8, 40-47], provided sufficient information to compare the highest and lowest categories of steps per day with death from all causes; however, there were not enough data to calculate mortality from CVD and cancer. Compared with the lowest categories of steps per day, the highest categories of steps per day were associated with a reduced risk of mortality in the overall sample.

Supplementary References

1. Mejborn, H., et al., *Dietary Intake of Red Meat, Processed Meat, and Poultry and Risk of Colorectal Cancer and All-Cause Mortality in the Context of Dietary Guideline Compliance.* Nutrients, 2020. **13**(1).
2. Sheehy, S., J.R. Palmer, and L. Rosenberg, *High Consumption of Red Meat Is Associated with Excess Mortality Among African-American Women.* J Nutr, 2020. **150**(12): p. 3249-3258.
3. Bellavia, A., F. Stilling, and A. Wolk, *High red meat intake and all-cause cardiovascular and cancer mortality: is the risk modified by fruit and vegetable intake?* Am J Clin Nutr, 2016. **104**(4): p. 1137-1143.
4. Rashmi Sinha, P.A.J.C., PhD; Barry I. Graubard, P D; and M. Michael F. Leitzmann, DrPH; Arthur Schatzkin, MD, DrPH, *Meat Intake and Mortality A Prospective Study of Over Half a Million People.* American Medical Association, 2009.
5. Dominguez, L.J., et al., *Should we recommend reductions in saturated fat intake or in red/processed meat consumption? The SUN prospective cohort study.* Clin Nutr, 2018. **37**(4): p. 1389-1398.
6. Yamamoto, N., et al., *Daily step count and all-cause mortality in a sample of Japanese elderly people: a cohort study.* BMC Public Health, 2018. **18**(1): p. 540.
7. Hansen, B.H., et al., *Step by step: Association of device-measured daily steps with all-cause mortality-A prospective cohort Study.* Scand J Med Sci Sports, 2020. **30**(9): p. 1705-1711.

8. Patel, A.V., et al., *Walking in Relation to Mortality in a Large Prospective Cohort of Older U.S. Adults.* Am J Prev Med, 2018. **54**(1): p. 10-19.
9. Alshahrani, S.M., et al., *Red and Processed Meat and Mortality in a Low Meat Intake Population.* Nutrients, 2019. **11**(3).
10. Argyridou, S., et al., *Relevance of physical function in the association of red and processed meat intake with all-cause, cardiovascular, and cancer mortality.* Nutr Metab Cardiovasc Dis, 2019. **29**(12): p. 1308-1315.
11. David Whiteman[1], John Muir[1], Lesley Jones[1], Mike Murphy[1] and Tim Key[2], *Dietary questions as determinants of mortality: the OXCHECK experience.* Public Health Nutrition, 1999.
12. Etemadi, A., et al., *Mortality from different causes associated with meat, heme iron, nitrates, and nitrites in the NIH-AARP Diet and Health Study: population based cohort study.* BMJ, 2017. **357**: p. j1957.
13. Farvid, M.S., et al., *Dietary Protein Sources and All-Cause and Cause-Specific Mortality: The Golestan Cohort Study in Iran.* Am J Prev Med, 2017. **52**(2): p. 237-248.
14. Iqbal, R., et al., *Associations of unprocessed and processed meat intake with mortality and cardiovascular disease in 21 countries [Prospective Urban Rural Epidemiology (PURE) Study]: a prospective cohort study.* Am J Clin Nutr, 2021. **114**(3): p. 1049-1058.
15. Kappeler, R., M. Eichholzer, and S. Rohrmann, *Meat consumption and diet quality and mortality in NHANES III.* Eur J Clin Nutr, 2013. **67**(6): p. 598-606.
16. Lee, J.E., et al., *Meat intake and cause-specific mortality: a pooled analysis of Asian prospective cohort studies.* Am J Clin Nutr, 2013. **98**(4): p. 1032-41.
17. Marjorie L. McCullough； Andrea S. Robertson, A.C. Eric J. Jacobs, Eugenia E. Calle, and, and M.J. Thun, *A Prospective Study of Diet and Stomach Cancer Mortality in United States Men and Women.* Cancer Epidemiology, Biomarkers & Prevention, 2001.
18. Pan, A., et al., *Red meat consumption and mortality: results from 2 prospective cohort studies.* Arch Intern Med, 2012. **172**(7): p. 555-63.
19. Rosalind A. Breslow1, Barry I. Graubard2, Rashmi Sinha3 & Amy F. Subar4, *Diet and lung cancer mortality: a 1987 National Health Interview Survey cohort study.* Cancer Causes and Control, 2000.
20. Sabine Rohrmann[1], Kim Overvad[3], H Bas Bueno-de-Mesquita[4,5], Marianne U Jakobsen[3], Rikke , et al., *Meat consumption and mortality - results from the European Prospective Investigation into Cancer and Nutrition.* BMC Medicine, 2013.
21. Saito, E., et al., *Association between meat intake and mortality due to all-cause and major causes of death in a Japanese population.* PLoS One, 2020. **15**(12): p. e0244007.
22. Segawa, H., et al., *Association of Red Meat Intake with the Risk of Cardiovascular Mortality in General Japanese Stratified by Kidney Function: NIPPON DATA80.* Nutrients, 2020. **12**(12).
23. Shaukat, A., et al., *BMI Is a Risk Factor for Colorectal Cancer Mortality.* Dig Dis Sci, 2017. **62**(9): p. 2511-2517.
24. Steven S. Coughlin, Eugenia E. Calle, Alpa V. Patel & Michael J. Thun, *Predictors of pancreatic cancer mortality among a large cohort of United States adults.* Cancer Causes

and Control, 2000.

25. Sun, Y., et al., *Association of Major Dietary Protein Sources With All-Cause and Cause-Specific Mortality: Prospective Cohort Study.* J Am Heart Assoc, 2021. **10**(5): p. e015553.

26. Takata, Y., et al., *Red meat and poultry intakes and risk of total and cause-specific mortality: results from cohort studies of Chinese adults in Shanghai.* PLoS One, 2013. **8**(2): p. e56963.

27. van den Brandt, P.A., *Red meat, processed meat, and other dietary protein sources and risk of overall and cause-specific mortality in The Netherlands Cohort Study.* Eur J Epidemiol, 2019. **34**(4): p. 351-369.

28. Zhang, J., et al., *Association of red and processed meat consumption with cardiovascular morbidity and mortality in participants with and without obesity: A prospective cohort study.* Clin Nutr, 2021. **40**(5): p. 3643-3649.

29. Zheng, Y., et al., *Association of changes in red meat consumption with total and cause specific mortality among US women and men: two prospective cohort studies.* BMJ, 2019. **365**: p. l2110.

30. Zhong, V.W., et al., *Associations of Processed Meat, Unprocessed Red Meat, Poultry, or Fish Intake With Incident Cardiovascular Disease and All-Cause Mortality.* JAMA Intern Med, 2020. **180**(4): p. 503-512.

31. Hsu, B., et al., *Total Physical Activity, Exercise Intensity, and Walking Speed as Predictors of All-Cause and Cause-Specific Mortality Over 7 Years in Older Men: The Concord Health and Aging in Men Project.* J Am Med Dir Assoc, 2018. **19**(3): p. 216-222.

32. Kamada, M., et al., *Strength Training and All-Cause, Cardiovascular Disease, and Cancer Mortality in Older Women: A Cohort Study.* J Am Heart Assoc, 2017. **6**(11).

33. Liu, Y., et al., *Associations of Resistance Exercise with Cardiovascular Disease Morbidity and Mortality.* Med Sci Sports Exerc, 2019. **51**(3): p. 499-508.

34. Patel, A.V., et al., *Relationship Between Muscle-Strengthening Activity and Cause-Specific Mortality in a Large US Cohort.* Prev Chronic Dis, 2020. **17**: p. E78.

35. Porter, A.K., C.C. Cuthbertson, and K.R. Evenson, *Participation in specific leisure-time activities and mortality risk among U.S. adults.* Ann Epidemiol, 2020. **50**: p. 27-34 e1.

36. Sheehan, C.M. and L. Li, *Associations of Exercise Types with All-Cause Mortality among U.S. Adults.* Med Sci Sports Exerc, 2020. **52**(12): p. 2554-2562.

37. Siahpush, M., et al., *Muscle-strengthening physical activity is associated with cancer mortality: results from the 1998-2011 National Health Interview Surveys, National Death Index record linkage.* Cancer Causes Control, 2019. **30**(6): p. 663-670.

38. Stamatakis, E., et al., *Does Strength-Promoting Exercise Confer Unique Health Benefits? A Pooled Analysis of Data on 11 Population Cohorts With All-Cause, Cancer, and Cardiovascular Mortality Endpoints.* Am J Epidemiol, 2018. **187**(5): p. 1102-1112.

39. Zhao, M., et al., *Recommended physical activity and all cause and cause specific mortality in US adults: prospective cohort study.* BMJ, 2020. **370**: p. m2031.

40. Dwyer, T., et al., *Objectively Measured Daily Steps and Subsequent Long Term All-Cause Mortality: The Tasped Prospective Cohort Study.* PLoS One, 2015. **10**(11): p. e0141274.

41. Jefferis, B.J., et al., *Objectively measured physical activity, sedentary behaviour and all-cause mortality in older men: does volume of activity matter more than pattern of*

accumulation? Br J Sports Med, 2019. **53**(16): p. 1013-1020.

42. Klenk, J., et al., *Objectively Measured Walking Duration and Sedentary Behaviour and Four-Year Mortality in Older People.* PLoS One, 2016. **11**(4): p. e0153779.
43. Lee, I.M., et al., *Association of Step Volume and Intensity With All-Cause Mortality in Older Women.* JAMA Intern Med, 2019. **179**(8): p. 1105-1112.
44. Manas, A., et al., *Association of accelerometer-derived step volume and intensity with hospitalizations and mortality in older adults: A prospective cohort study.* J Sport Health Sci, 2021.
45. Oftedal, S., et al., *Daily steps and diet, but not sleep, are related to mortality in older Australians.* J Sci Med Sport, 2020. **23**(3): p. 276-282.
46. Paluch, A.E., et al., *Steps per Day and All-Cause Mortality in Middle-aged Adults in the Coronary Artery Risk Development in Young Adults Study.* JAMA Netw Open, 2021. **4**(9): p. e2124516.
47. Saint-Maurice, P.F., et al., *Association of Daily Step Count and Step Intensity With Mortality Among US Adults.* JAMA, 2020. **323**(12): p. 1151-1160.

Supplementary Tables

Table S1. Medical subject headings (MeSH) and non-MeSH keywords used to search relevant publications.

stage 1：	(((red meat) OR (processed meat)) OR (meat products)) OR (Meat consumption) AND ((All-cause mortality) OR (Cardiovascular disease mortality)) OR (Cancer mortality)
stage 2：	((((step/day) OR (steps/day)) OR (step counts/day)) OR (step count/day)) OR (walking) AND ((All-cause mortality) OR (Cardiovascular disease mortality)) OR (Cancer mortality)
stage 3：	((((((((((((("resistance train*") OR ("resistance exercise*")) OR ("strength train*")) OR ("strength exercise*")) OR ("strengthening programs")) OR ("weight bearing exercise")) OR ("weight exercise*")) OR ("weight train*")) OR ("circuit training")) OR ("isometric exercise*")) OR ("strength endurance*")) OR ("weight bearing strengthening")) OR ("weight lifting")) AND ((All-cause mortality) OR (Cardiovascular disease mortality)) OR (Cancer mortality)

Table S2. Characteristics of included studies for association between meat intake and mortality from cardiovascular disease and cancer.

Author, Year of Publication, Country	Age*	No of participants	Follow-up (years) †	No of deaths	Exposure	Exposure assessment	Comparison for meat intake	Effect size (95% CI) §	Adjustment¶
**CVD mortality**									
Piet A, 2019,	55-69	M 58279	10	5797	Red meat	FFQ	140.4 vs 41.3g/day	HR 0.95(0.77-1.17)	1,2,4,5,6,9,11,14,19,
Netherland		W 62573		3026	Processed		30.8 vs 0g/day	HR 1.26(1.01-1.56)	20,21,22,23,24,25,26,27
Alshahrani,	＞25	72149	11.8	7961	Red meat	FFQ	41.7vs4g/day	HR 1.26(1.05-1.5)	1,2,3,4,5,6,9,18,21,22,
2019, US					Processed		9.4vs0.7g/day	HR 1.12(0.93-1.36)	24,32,33,34,35,36,37,
and Canada					Combined		42.8vs1.4g/day	HR 1.34(1.05-1.5)	38,39,40,41,42,43,44,45,4647,48
Argyridou, 2019, UK	40-69	419075	7	15058	Combined	FFQ	7.0vs1.5 servings/ week	HR1.238(1.062-1.442)	1,2,3,4,6,8,9,11,14,24,47,48,49,50,51,52
Sheehy,	38	W 56314	22	5054	Red meat	FFQ	1.0vs0.01serving/day	HR 1.62(1.33-1.97)	4,5,6,8,21,24,28,30,60,
2020, US					Processed		1.2vs0.01serving/day	HR 1.66(1.38-2.00)	61,62
Saito,	45-74	M 40072	14	6266	Red meat	FFQ	92.9vs14.3g/d	HR 1.36(0.95-1.95)	1,5,6,9,11,14,15,16,18,
2020, Japan					Processed		8.4vs1.3g/d	HR 0.85(0.64-1.13)	21,22,24,47,63,64,65

		W 47435		3620	Red meat		90.3vs13.6g/d	HR 0.62(0.41-0.95)	
					Processed		11.7vs2.1g/d	HR 1.01(0.71-1.42)	
Sinha,	50-71	500000	10	M 47976	Red meat	124-item	68.1vs9.3g/1000kcal	HR 1.27(1.20-1.35)	4,6,8,9,11,14,19,24,26
2009, US					Processed	FFQ	19.4vs5.1 g/1000kcal	HR 1.09(1.03-1.15)	30,37,66
				W 23276	Red meat		65.9vs9.1 g/1000kcal	HR 1.50(1.37-1.65)	
					Processed		16.0vs3.8 g/1000kcal	HR 1.38(1.26-1.51)	
Pan,	NA	M 37698	22	M 8926	Red meat	FFQ	2.36vs0.22 servings/day	HR 1.32(1.16-1.49)	1,3,5,6,8,9,11,14,21,22,
2012, US					Processed		2.36vs0.22 servings/day	HR 1.25(1.11-1.41)	24,28,29,30,34,35,36,43
					Combined		2.36vs0.22 servings/day	HR 1.35(1.19-1.53)	
		W 83644	28	W 15000	Red meat		3.1vs0.53servings/day	HR 1.39(1.24-1.55)	
					Processed		3.1vs0.53servings/day	HR 1.29(1.15-1.43)	
					Combined		3.1vs0.53servings/day	HR 1.45(1.30-1.63)	
Takata,	40-74	M 61128	334281**	2733	Red meat	FFQ	114.9vs20.0g/day	HR 1.15(0.90-1.48)	1,4,5,6,7,8,9,11,14,47,
2013, China		W 73162	803265**	4210			94.8vs15.0g/day	HR 0.89(0.72-1.09)	48,59,67,68,69

Segawa, 2020,	≥30	M 3986	29	532	Red meat	Standard Tables	91.1vs20.1g/day	HR 1.08(0.88-1.33)	1,6,9,11,14,22,24,52,70,
Japan		W 5126		585	Red meat	for Foods in Japan	15.2vs73.4g/day	HR 1.01(0.83-1.24)	71
Bellavia, 2016,	45-83	M 40089	16	10423	Red meat	FFQ	140vs31g/day	HR 1.29(1.14-1.46)	2,4,5,7,8,9,22,24,47
Sweden		W 34556		7486					
Zhang, 2021, UK	56	428070	7.2	1837	Combined	touchscreen questionnaire	6.0vs2.0servings/week	HR 1.33(1.19-1.49)	1,2,3,4,6,9,72
Kappeler, 2013,	＞18	17611	22	M 1908	Red meat	FFQ	45+vs0-6times/week	HR 0.76(0.26-2.23)	1,2,3,6,8,9,11,14,21,22,
US					Processed		45+vs0-6times/week	HR 0.74(0.41-1.33)	24,26,29,32,35,36,37,61
				W 1775	Red meat		45+vs0-6times/week	HR 3.50(1.35-9.05)	73,74
					Processed		45+vs0-6times/week	HR 1.01(0.67-1.52)	
Lee, 2013,	17-92	M 112310	6.6-15.5	23515	Red meat	FFQ	High/low	HR 0.87 (0.78, 0.98)	1,4,5,6,9,11,14,24,76
Asian		W 184411		16699	Red meat		High/low	HR 1.03 (0.85, 1.25)	
Farvid, 2016, Iran	51.6	42403	11	3291	Red meat	FFQ	0.43vs0.02serving/day	HR 1.07(0.90-1.28)	1,2,3,4,5,6,8,9,24,30,37,51,70,76
Iqbal, 2021,	35-70	134297	9.5	7789	Red meat	FFQ	≥250/＜50g/week	HR 0.97 (0.84, 1.14)	1,2,4,5,6,8,11,12,14,41,

21 countries					Processed		≥250/＜50g/week	HR 1.39 (0.73, 2.63)	22,78,79,80
Sun, 2021,	50-79	102521	18.1	25976	Red meat	FFQ	3.2/0.3oz equivalent/d	HR 1.12(1.02-1.23)	1,3,4,5,6,8,9,11,14,17
US					Processed		1.0/0.01oz equivalent/d	HR 0.99(0.91-1.07)	22,41,43,67,71,74,81
					Combined		3.9/0.4oz equivalent/d	HR 1.14(1.04-1.25)	
**Cancer mortality**									
Piet A, 2019,	55-69	M 58279	10	5797	Red meat	FFQ	140.4 vs 41.3g/day	HR 1.02(0.85-1.21)	1,2,4,5,6,9,11,14,19,20,
Netherland		W 62573		3026	Processed		30.8 vs 0g/day	HR 1.16(0.97-1.39)	21,22,23,24,25,26,27
Alshahrani,	＞25	72149	11.8	7961	Red meat	FFQ	41.7vs4g/day	HR 1.04(0.85-1.27)	1,2,3,4,5,6,9,18,21,22,
2019, US					Processed		9.4vs0.7g/day	HR 1.01(0.83-1.23)	24,32,33,34,35,36,37,38
and Canada					Combined		42.8vs1.4g/day	HR 1.00(0.85-1.22)	39,40,41,42,43,44,45,4647,48
Argyridou, 2019, UK	40-69	419075	7	15058	Combined	FFQ	7.0vs1.5 servings/week	HR 1.183(1.082-1.293)	1,2,3,4,6,8,9,11,14,24, 47,48,49,50,51,52
Etemadi,	50-71	M 316505	16	84848	Red meat	124-item	50.3vs6.9g/1000kcal	HR 1.13(1.09-1.17)	1,2,3,4,5,6,8,9,11,14,24,
2017, US		W 220464		43676	Processed	FFQ	17.2vs2.3g/1000kcal	HR 1.13(1.09-1.17)	29,30,37,54,55,56,57,58

Sheehy, 2020, US	38	W 56314	22	5054	Red meat	FFQ	1.0vs0.01serving/day	HR 1.20(1.01-1.42)	4,5,6,8,21,24,28,30,60, 61,62
					Processed		1.2vs0.01serving/day	HR 1.09(0.93-1.28)	
Saito, 2020, Japan	45-74	M 40072	14	6266	Red meat	FFQ	92.9vs14.3g/d	HR 1.07(0.91-1.25)	1,5,6,9,11,14,15,16,18, 21,22,24,47,63,64,65
					Processed		8.4vs1.3g/d	HR 1.00(0.88-1.13)	
		W 47435		3620	Red meat		90.3vs13.6g/d	HR 1.23(0.99-1.51)	
					Processed		11.7vs2.1g/d	HR 1.10(0.93-1.30)	
Sinha, 2009, US	50-71	500000	10	M 47976	Red meat	124-item FFQ	68.1vs9.3g/1000kcal	HR 1.22(1.16-1.29)	4,6,8,9,11,14,19,24,26 30,37,66
					Processed		19.4vs5.1 g/1000kcal	HR 1.12(1.06-1.19)	
				W 23276	Red meat		65.9vs9.1 g/1000kcal	HR 1.20(1.12-1.30)	
					Processed		16.0vs3.8 g/1000kcal	HR 1.11(1.04-1.19)	
Pan, 2012, US	NA	M 37698	22	M 8926	Red meat	FFQ	2.36vs0.22 servings/day	HR 1.18(1.05-1.33)	1,3,5,6,8,9,11,14,21,22, 24,28,29,30,34,35,36,43
					Processed		2.36vs0.22 servings/day	HR 1.15(1.02-1.29)	
					Combined		2.36vs0.22 servings/day	HR 1.24(1.09-1.40)	
		W 83644	28	W 15000	Red meat		3.1vs0.53servings/day	HR 1.17(1.08-1.27)	

					Processed		3.1vs0.53servings/day	HR 1.14(1.05-1.23)	
					Combined		3.1vs0.53servings/day	HR 1.17(1.08-1.28)	
Takata, 2013, China	40-74	M 61483	334281**	2733	Red meat	FFQ	114.9vs20.0g/day	HR 1.17(0.95-1.44)	1,4,5,6,7,8,9,11,14,47, 48,59,67,68,69
		W 74941	803265**	4210	Red meat		94.8vs15.0g/day	HR 0.92(0.78-1.09)	
		T：136424	1137546	6943	Red meat		104.85vs17.5g/day	HR 1.01(0.89-1.16)	
Bellavia, 2016, Sweden	45-83	M 40089	16	10423	Red meat	FFQ	140vs31g/day	HR 1.00(0.88-1.43)	2,4,5,7,8,9,22,24,47
		W 34556		7486					
Breslow, 2000, US	18-87	20195	8.5	158	Red meat	59-item FFQ	9.0vs1.4servings/week	RR 1.6(1.0-2.06)	1,2,6,7,19
					Processed		4.5vs0servings/week	RR 0.8(0.5-1.4)	
Kappeler, 2013, US	＞18	17611	22	M 1908	Red meat	FFQ	45+vs0-6times/week	HR 0.76(0.26-2.23)	1,2,3,6,8,9,11,14,21,22, 24,26,29,32,35,36,37,61 73,74
					Processed		45+vs0-6times/week	HR 1.31(0.84-2.05)	
				W 1775	Red meat		45+vs0-6times/week	HR 0.63(0.09-4.69)	
					Processed		45+vs0-6times/week	HR 0.99(0.45-2.17)	
Lee, 2013,	17-92	M 112310	6.6-15.5	23515	Red meat	FFQ	High/low	HR 0.90 (0.77, 1.05)	1,4,5,6,9,11,14,24,76

Asian		W 184411		16699	Red meat		High/low	HR 0.85 (0.76, 0.94)	
Whiteman,	35-64	10522	9	514	Red meat	FFQ	4-7vs＜1day week-1	HR 0.88(0.59-1.32)	1,2,6
1999, UK					Processed		4-7vs＜1day week-1	HR 1.22(0.60-2.51)	
Coughlin, 2000,	45-71	M 483109	14	1967	Red meat	FFQ		RR 1.1(0.9-1.2)	1,3,4,6,9,11,14,22,24,30
US	43-71	W 619199		1784	Red meat			RR 0.9(1.08-1.0)	,59,75
McCullough, 2001,	NR	M 436654	14	910	Processed	FFQ	4.5+vs＜1times/week	RR 1.08(0.87-1.33)	1,3,4,6,24,32,33
US		W 533391		439	Processed		3+vs＜1.5times/week	RR 1.11(0.88-1.39)	
Farvid, 2016, Iran	51.6	42403	11	3291	Red meat	FFQ	0.43vs0.02serving/day	HR 1.03(0.82-1.30)	1,2,3,4,5,6,8,9,24,30,37,51,70,76
Shaukat, 2017, US	50-80	46551	30	732	Processed	FFQ	NR	HR 1.37(0.74-2.59)	NR
Iqbal, 2021,	35-70	164007	9.5	7789	Red meat	FFQ	≥250/＜50g/week	HR 0.90 (0.76, 1.05)	1,2,4,5,6,8,11,12,14,41,
21 countries					Processed		≥250/＜50g/week	HR 1.84 (1.14, 2.97)	22,78,79,80
Sun, 2021,	50-79	102521	18.1	25976	Red meat	FFQ	3.2/0.3oz equivalent/d	HR 0.98(0.90-1.08)	1,3,4,5,6,8,9,11,14,17
US					Processed		1.0/0.01oz	HR 1.0(0.92-1.08)	22,41,43,67,71,74,81

		equivalent/d	
	Combined	3.9/0.4oz equivalent/d	HR 1.01(0.92-1.10)

FFQ=food frequency questionnaire; HR=hazard ratio; M=men; RR=risk ratio; CI=confidence interval; NR=not reported; W=women.

*Presented as mean or range.

**Person-years.

†Number of years that individuals were followed up in the prospective cohort studies.

§These effect sizes are for comparison of the highest and the lowest categories.

¶Adjustments: age (1), sex (2), race/ethnicity (3), educational level (4), total energy (5), smoking status (6), smoking pack-years (7), physical activity (8), alcohol intake (9), hormone therapy (10), fruits (11), legumes (12), potatoes (13), vegetables (14), low-fat dairy products (15), high-fat dairy products (16), sugar-sweetened beverages (17), eggs (18), number of cigarettes smoked per day (19), years of smoking (20), history of physician-diagnosed hypertension (21), history of physician-diagnosed diabetes (22), body height (23), BMI (24), non-occupational physical activity (25), use of nutritional supplements (26), in women postmenopausal HRT (27), family history of myocardial infarction (28), family history of diabetes (29), family history of cancer (30), weight (31), aspirin use (32), multivitamin use (33), menopausal status (34), postmeno pausal hormone therapy use for women (35), physician diagnosed hypercholesterolemia (36), marital status (37), exercise (38), sleep (39), the use of statin (40), the use of blood pressure medications (41), Cruciferous vegetables (42), whole grain (43), nuts (44), seeds (45), total dairy (46),fish (47), unprocessed poultry (48), Townsend score (49), employment (50), number of medications (51), salt added to food (52), time TV viewing (53), quintiles of a composite deprivation index (54), perceived health at baseline (55), history of heart disease (56), history of stroke (57), usual activity throughout the day (58), total meat intake (59), geographic region (60), neighborhood SES (socioeconomic status) (61), Alternative Healthy Eating Index without red meat (62), quartile of metabolic equivalent task-hours/d (63), sodium (64), total fat (65), time since quitting for former smokers (66), income (67), occupation (68), comorbidity index (69), systolic blood pressure (70), proteinuria (71), overall health (72), use of ibuprofen (73), family history of hypercholesterolemia (74), history of gallstones (75), residency (76), opium use (77), wealth score (78), location (79), starchy foods (80), unopposed estrogen use (81), year of entering the cohort (82), history of depression (83), following special diets at baseline (84).

Table S3. Characteristics of included studies for association between muscle-strengthening activity and mortality from cardiovascular disease and cancer.

Author, Year of Publication, Country	Age*	Sample size	Follow-up (years) †	No of cases	Exposure	Exposure assessment	Comparison for meat intake	Effect size (95% CI) §	Adjustment¶
**CVD mortality**									
Zhao, 2020, US	⩾18	479856	8.75	59819	MSA	self-reported	<2 times/week	1	1,2,3,4,5,6,7,8,9
							⩾2 times/week	0.82 (0.74-0.92)	
Stamatakis, 2017, UK	≥19	72459	9.2	5763	Strength-promoting exercise	questionnaire	None	1	1,2,4,6,7,8,11,12
							Any	0.88 (0.71-1.08)	
Kamada, 2017, US	62.2 (Mean)	28879	12	3055	Strength training	questionnaire	0	1	2,3,4,6,7,8,13,14,15,16, 17,18,19,20,21,22,23,24,25,26,27,28
							⩾150min/week	0.72 (0.42–1.22)	
LIU, 2018, US	18-89	12591	10	276	Resistance exercise	questionnaire	0	1	1,2,6,7,8,24,27,29,30
							⩾120min/week	0.96 (0.45–2.05)	
Patel,	50-74	72462	13	17750	MSA	questionnaire	0	1	1,2,4,5,6,7,8,31,32,33,

2020, US							⩾2h/week	1.03 (0.90-1.19)	34,35,36,37
Porter, 2020, US	46.3 (Mean)	17938	11.9	3799	Weightlifting	questionnaire	No	1	1,2,3,4,6,7,8,38
							Yes	0.53 (0.21, 1.29)	
Hsu, 2017, Australian	⩾70	1705	7	519	Muscle strengthening exercise	questionnaire	No	1	2,3,4,6,7,8,26,27,36,50, 51,52,53
							Yes	0.53 (0.27-1.04)	
**Cancer mortality**									
Siahpush, 2018, US	⩾18	310628	7.9	7275	MSA	questionnaire	No	1	1,2,3,4,5,6,7,9,28,32, 37,54
							Yes	0.81 (0.73-0.89)	
Zhao, 2020, US	⩾18	479856	8.75	59819	MSA	self-reported	<2 times/week	1	1,2,3,4,5,6,7,8,9
							⩾2 times/week	0.85 (0.77-0.95)	
Stamatakis, 2017, UK	≥19	72459	9.2	5763	Strength-promoting exercise	questionnaire	None	1	1,2,4,6,7,8,11,12
							Any	0.69（0.57-0.84）	
Kamada, 2017, US	62.2 (Mean)	28879	12	3055	Strength training	questionnaire	0	1	2,3,4,6,7,8,13,14,15,16, 17,18,19,20,21,22,23,24,25,26,27,28
							⩾150min/week	1.10 (0.77–1.56)	

Patel, 2020, US	50-74	72462	13	17750	MSA	questionnaire	0	1	1,2,4,5,6,7,8,31,32,33, 34,35,36,37
							⩾2h/week	1.02 (0.89–1.17)	
Porter, 2020, US	46.3 (Mean)	17938	11.9	3799	Weightlifting	questionnaire	No	1	1,2,3,4,6,7,8,38
							Yes	0.89 (0.67-1.17)	
Hsu, 2017, Australian	⩾70	1705	7	519	Muscle strengthening exercise	questionnaire	No	1	2,3,4,6,7,8,26,27,36,50, 51,52,53
							Yes	0.72 (0.49-1.06)	

MSA=muscle strengthening activity; HR=hazard ratio; RR=risk ratio; CI=confidence interval; CVD=cardiovascular disease; NR=not reported;

*Presented as mean or range.

**Person-years.

† The number of years that individuals were followed up in the prospective cohort studies.

§These effect sizes are for comparison of the highest and the lowest categories.

¶Adjustments: Sex(1), age (2), race/ethnicity (3), education (4), marital status (5), body mass index (6), smoking status (7), alcohol intake (8), chronic conditions (9), long-standing illness (10), psychological distress (11), weekly physical activity volume excluding the volume of strength-promoting activity (12), trial randomization (13), postmenopausal status (14), hormone use (15), parental history of myocardial infarction or cancer (16), energy intake (17), saturated fat intake (18), fiber intake (19), fruit and vegetable intake (20), physical examination for screening (21), time per week spent in aerobic (22), MVPA (for strength training and vice versa) (23), incidence of hypertension (24), high cholesterol (25), cardiovascular diseases (26), diabetes mellitus (27), cancer before and during follow-up (28), parental history of CVD (29), hypercholesterolemia (30), survey type (31), self-reported overall health (32), work status (33), TV sitting time (34), aspirin use (35), comorbidity score (36), aerobic moderate-to-vigorous physical activity (37), household (38), use of an

ambulatory device (39), total cholesterol level (40), statin medication use (41), measured mean arterial blood pressure (42), the following physician diagnosed conditions (43), arthritis (44), stroke (45), serum C-reactive protein (46), congestive heart failure (47), coronary artery disease (48), emphysema (49), self-rated health (50), ADL disability (51), depression (52), PASE score (53), income (54), nativity status (55), census region of residence (56), home ownership (57), eGFR (58), need special equipment to walk (59).

Table S4: Results of risk of bias assessment based on the Newcastle-Ottawa scale.

Study ID	Selection				Comparability	Outcome			Quality rating
	Representativeness of the exposed cohort	Selection of the Non-exposed cohort	Ascertainment of exposure	Demonstration that outcome of interest was not present at start of study	Comparability of cohorts on the basis of the design or analysis	Assessment of outcome	Was follow-up long enough for outcomes to occur	Adequacy of follow up of cohorts	
Zhong et al, 2019	☆	☆	☆	☆	☆	☆	☆	☆	8
Piet A et al, 2019	☆	☆	☆	☆	☆	☆	☆	☆	8
Zheng et al, 2019		☆	☆	☆	☆	☆	☆	☆	7
Alshahrani et al, 2019	☆	☆	☆	☆	☆	☆	☆	☆	8
Mejborn et al, 2020	☆	☆	☆	☆	☆	☆	☆	☆	8
Argyridou et al, 2019	☆	☆	☆	☆	☆	☆	☆	☆	8
Dominguez et al, 2017	☆	☆	☆	☆	☆	☆	☆	☆	8
Etemadi et al, 2017	☆	☆	☆	☆	☆	☆	☆	☆	8
Sheehy et al, 2020		☆	☆	☆	☆	☆	☆	☆	7
Saito et al, 2020	☆	☆	☆	☆	☆	☆	☆	☆	8

Rohrmann et al, 2013	☆	☆	☆	☆	☆	☆	☆	☆	8
Sinha et al, 2009	☆	☆	☆	☆	☆	☆	☆	☆	8
An Pan et al, 2012	☆	☆	☆	☆	☆	☆	☆	☆	8
takata et al, 2013	☆	☆	☆	☆	☆	☆	☆	☆	8
Segawa et al, 2020	☆	☆	☆	☆	☆	☆	☆	☆	8
Bellavia et al, 2016	☆	☆	☆	☆	☆	☆	☆	☆	8
Breslow et al, 2000	☆	☆	☆	☆	☆	☆	☆	☆	8
Zhang et al,2021	☆	☆		☆	☆	☆	☆	☆	7
Kappeler et al,2013	☆	☆	☆	☆	☆	☆	☆	☆	8
Lee et al, 2013	☆	☆	☆	☆	☆	☆	☆	☆	8
Whiteman et al,1999	☆	☆		☆	☆	☆	☆	☆	7
McCullough et al,2001	☆	☆		☆	☆	☆	☆	☆	7
Farvid et al,2016	☆	☆	☆	☆	☆	☆	☆	☆	8
Shaukat et al,2017	☆	☆		☆	☆	☆	☆	☆	7
Iqbal et al,2021	☆	☆	☆	☆	☆	☆	☆	☆	8
Sun et al, 2021		☆	☆	☆	☆	☆	☆	☆	7
Coughlin et al, 2000	☆	☆		☆	☆	☆	☆	☆	7

Lee et al, 2019	☆	☆	☆	☆	☆	☆	☆	☆	8
Hansen et al, 2020	☆	☆	☆	☆	☆	☆	☆	☆	8
Maurice et al, 2020	☆	☆	☆	☆	☆	☆	☆	☆	8
Jefferis et al, 2017		☆	☆	☆	☆	☆	☆	☆	7
Yamamoto et al, 2018	☆	☆	☆	☆	☆		☆	☆	7
Oftedal et al, 2019	☆	☆	☆	☆	☆	☆	☆	☆	8
Dwyer et al, 2015	☆	☆	☆	☆	☆	☆	☆	☆	8
Paluch et al,2021	☆	☆	☆	☆	☆	☆	☆	☆	8
Manas et al, 2021		☆	☆	☆	☆	☆		☆	6
Klenk et al, 2016	☆	☆	☆	☆	☆	☆	☆	☆	8
Patel et al, 2017	☆	☆		☆	☆	☆	☆	☆	7
Siahpush et al, 2018	☆	☆		☆	☆	☆	☆	☆	7
Zhao et al, 2020	☆	☆		☆	☆	☆	☆	☆	7
Stamatakis et al, 2017	☆	☆	☆	☆	☆	☆	☆	☆	8
Kamada et al, 2017		☆	☆	☆	☆	☆	☆	☆	7
Liu et al, 2018	☆	☆		☆	☆	☆	☆	☆	7
Patel et al, 2020		☆		☆	☆	☆	☆	☆	6

Porter et al, 2020	☆	☆	☆	☆	☆	☆	☆	☆	8
Hsu et al, 2017		☆	☆	☆	☆	☆	☆		6
Sheehan et al, 2020	☆	☆		☆	☆	☆	☆	☆	7

Table S5. Association of meat intake, daily steps and muscle-strengthening activity for different subgroups.

Relationship	Grouping factors	Group standard	No. of studies	P-value	I^2	ES(95%CI)
**Red meat intake and all-cause mortality**	Region	European	5	0.002	76.0	0.97(0.78-1.16)
		America	11	0.000	90.5	1.22(1.16-1.28)
		Asia	5	0.024	64.3	1.01(0.92-1.09)
		Across the region	1	-	-	-
	Exposure investigation	FFQ	18	0.000	90.8%	1.16(1.10-1.23)
		Not FFQ	4	0.001	81.9%	0.91(0.78-1.04)
	No. of participants	> 100 thousand	12	0.000	94.9%	1.12(1.04-1.20)
		< 100 thousand	10	0.000	81.6%	1.11(1.00-1.22)
	Exposure evaluation	gram	14	0.000	94.1%	1.09(1.01-1.17)
		Serving	6	0.000	82.4%	1.23(1.13-1.32)
		timing	2	0.026	79.9%	0.98(0.35-1.61)
	Published year	After 2010	19	0.000	88.5%	1.10(1.04-1.17)
		Before 2010	3	0.000	95.3%	1.16(0.96-1.36)
**Red meat intake and CVD mortality**	Region	European	1	-	-	-
		America	8	0.001	72.7%	1.33(1.22-1.44)
		Asia	7	0.042	54.0%	0.96(0.85-1.07)
		Across the region	1	-	-	-
	Exposure investigation	FFQ	15	0.000	87.6%	1.16(1.04-1.29)
		Not FFQ	2	0.652	0.0%	1.04(0.89-1.19)
	No. of participants	> 100 thousand	10	0.000	89.8%	1.16(1.02-1.29)
		< 100 thousand	7	0.000	76.4%	1.14(0.90-1.39)
	Exposure evaluation	gram	13	0.000	86.8%	1.08(0.96-1.20)
		Serving	3	0.263	25.1%	1.39(1.27-1.52)
		timing	1	-	-	-

	Published year	After 2010	15	0.000	79.9%	1.11(0.99-1.22)
		Before 2010	2	0.005	87.6%	1.38(1.15-1.60)
**Red meat intake and cancer mortality**	Region	European	3	0.252	27.4%	1.06(0.90-1.23)
		America	12	0.000	74.2%	1.12(1.05-1.18)
		Asia	5	0.016	67.2%	0.98(0.87-1.10)
		Across the region	1	-	-	-
	Exposure investigation	FFQ	18	0.000	78.5%	1.08(1.01-1.14)
		Not FFQ	3	0.086	59.3%	0.97(0.82-1.13)
	No. of participants	> 100 thousand	14	0.000	84.4%	1.05(0.98-1.13)
		< 100 thousand	7	0.485	0.0%	1.11(1.01-1.21)
	Exposure evaluation	gram	13	0.000	83.3%	1.05(0.98-1.13)
		Serving	4	0.768	0.0%	1.18(1.11-1.25)
		timing	2	0.868	0.0%	0.87(0.54-1.19)
	Published year	After 2010	15	0.000	76.3%	1.05(0.98-1.12)
		Before 2010	6	0.000	84.1%	1.10(0.98-1.24)
**Processed meat intake and all-cause mortality**	Region	European	5	0.090	50.3%	1.22(1.02-1.41)
		America	11	0.000	81.3%	1.17(1.13-1.22)
		Asia	2	0.313	1.7%	1.00(0.94-1.07)
		Across the region	1	-	-	-
	Exposure investigation	FFQ	17	0.000	81.1%	1.16(1.12-1.21)
		Not FFQ	2	0.923	0.0%	1.02(0.82-1.23)
	No. of participants	> 100 thousand	9	0.000	83.0%	1.19(1.14-1.24)
		< 100 thousand	10	0.000	70.9%	1.11(1.03-1.20)
	Exposure evaluation	gram	11	0.000	83.1%	1.14(1.09-1.19)
		Serving	6	0.001	74.8%	1.21(1.13-1.30)
		timing	2	0.975	0.0%	1.06(0.84-1.28)
	Published year	After 2010	16	0.000	79.0%	1.15(1.10-1.20)

		Before 2010	3	0.031	71.3%	1.20(1.12-1.28)
**Processed meat intake and CVD mortality**	Region	European	2	0.117	59.3%	1.44(1.00-1.88)
		America	8	0.000	86.1%	1.21(1.08-1.33)
		Asia	2	0.467	0.0%	0.90(0.70-1.10)
		Across the region	1	-	-	-
	Exposure investigation	FFQ	13	-	-	-
		Not FFQ	0	-	-	-
	No. of participants	> 100 thousand	8	0.000	83.6%	1.23(1.10-1.35)
		< 100 thousand	5	0.001	79.2%	1.11(0.83-1.40)
	Exposure evaluation	gram	9	0.000	79.4%	1.15(1.02-1.28)
		serving	3	0.061	64.2%	1.35(1.17-1.53)
		timing	1	-	-	-
	Published year	After 2010	11	0.000	77.6%	1.19(1.04-1.33)
		Before 2010	2	0.000	94.0%	1.23(0.95-1.51)
**Mixed meat intake and all-cause mortality**	Region	European	3	0.759	0.0%	1.23(1.17-1.29)
		America	5	0.000	92.4%	1.22(1.12-1.33)
		Asia	3	0.000	88.3%	1.22(1.06-1.38)
	Exposure investigation	FFQ	10	0.000	85.8%	1.24(1.18-1.30)
		Not FFQ	1	-	-	-
	No. of participants	> 100 thousand	5	0.000	82.0%	1.21(1.09-1.32)
		< 100 thousand	6	0.000	89.4%	1.24(1.16-1.32)
	Exposure evaluation	gram	4	0.000	85.9%	1.20(1.11-1.30)
		serving	7	0.000	89.7%	1.24(1.14-1.34)
		timing	0	-	-	-
	Published year	After 2010	11	-	-	-
		Before 2010	0	-	-	-
**Mixed meat**	Region	European	3	0.757	0.0%	1.29(1.20-1.39)
		America	4	0.000	83.1%	1.38(1.17-1.59)

**intake and**		Asia	1	-	-	-
**CVD**	Exposure investigation	FFQ	6	0.003	72.2%	1.33(1.20-1.46)
**mortality**		Not FFQ	2	0.035	77.4%	1.21(0.95-1.46)
	No. of participants	> 100 thousand	6	0.008	68.2%	1.26(1.15-1.38)
		< 100 thousand	2	0.028	79.2%	1.47(1.07-1.87)
	Exposure evaluation	gram	2	0.010	66.6%	1.33(1.20-1.47)
		serving	6	0.124	57.6%	1.20(1.19-1.40)
		timing	0	-	-	-
	Published year	After 2010	8	-	-	-
		Before 2010	0	-	-	-
**Daily steps**	Region	European	3	0.000	93.7%	0.55(0.12-0.97)
**and**		America	4	0.000	96.8%	0.51(0.21-0.81)
**all-cause**		Asia	1	-	-	-
**mortality**		Australia	2	0.760	0.0%	0.94(0.90-0.97)
	Exposure investigation	Accelerometer	6	0.000	95.1%	0.48(0.21-0.75)
		pedometer	3	0.041	68.8%	0.92(0.85-0.99)
		by asking	1	-	-	-
	No. of participants	> 2000 thousand	5	0.000	94.8%	0.62(0.50-0.81)
		< 2000 thousand	5	0.000	97.3%	0.62(0.36-0.88)
	Exposure evaluation	Steps	8	0.000	96.6%	0.62(0.45-0.80)
		Walking duration	2	0.028	79.3%	0.64(0.51-0.76)
**MSA**	Region	US	6	0.168	35.9%	0.94(0.88-1.00)
**and**		Not US	2	0.743	0.0%	0.77(0.68-0.85)
**all-cause**	No. of participants	> 3000 thousand	5	0.064	54.9%	0.93(0.86-1.01)
**mortality**		< 3000 thousand	3	0.208	36.3%	0.84(0.69-0.99)
	Followed-up years	< 10	3	0.040	69.0%	0.82(0.72-0.93)

		> 10	5	0.797	0.0%	0.99(0.92-1.05)
	Exposure evaluation	Time	5	0.002	75.9%	0.91(0.80-1.01)
		No/yes	3	0.329	10.1%	0.91(0.80-1.02)
**MSA and CVD mortality**	Region	US	5	0.093	49.8%	0.87(0.71-1.02)
		Not US	2	0.108	61.2%	0.75(0.41-1.08)
	No. of participants	> 3000 thousand	4	0.031	66.3%	0.86(0.67-1.04)
		< 3000 thousand	3	0.417	0.0%	0.82(0.66-0.98)
	Follow-up years	< 10	3	0.275	22.6%	0.81(0.71-0.92)
		> 10	4	0.195	36.2%	0.88(0.63-1.12)
	Exposure evaluation	Time	5	0.168	38.0%	0.89(0.78-1.00)
		No/yes	2	1.000	0.0%	0.53(0.22-0.84)

Table S6: The balanced relationship between meat intake and physical activity (all-cause mortality).

All-cause mortality							
Red meat(g/day)	Processed meat(g/day)	Mixed meat(g/day)	Increased risk of mortality(%)	MSA(mins/week)	MSA(mins/week)	Daily steps(steps/day)	Reduced risk of mortality (%)
0	0	0	0	0	145.27	1895	0
32.38	13.73	25.44	5	8.04	126.47	2394.99	5
53.30	21.56	49.57	10	16.5	107.67	2975.67	10
74.07	30.93	73.71	15	25.6	90.58	3548.90	15
**103.40**	**50**	**112.50**	**20**	**39.5**	**61.52**	**4100.00**	**20**
128.23	58.13	139.27	25	-	-	4777.27	25
163.18	82.53	191.08	30	-	-	5432.40	30
196.32	106.94	325.94	35	-	-	6087.53	35
-	129.60	-	40	-	-	6824.55	40
-	154.01	-	45	-	-	7971.02	45
-	178.42	-	50	-	-	11815.00	50
-	199.95	-	55				

Table S7: The balanced relationship between meat intake and physical activity (CVD mortality).

CVD mortality						
Red meat(g/day)	Processed meat(g/day)	Mixed meat(g/day)	Increased risk of mortality(%)	MSA(mins/ week)	MSA(mins/ week)	Reduced risk of mortality (%)
0	0	0	0	0	131.76	0
43.44	37.41	34.83	5	7.15	92.07	5
59.18	51.28	72.49	10	14.30	72.66	10
76.67	72.13	113.83	15	21.30	52.96	15
97.66	105.38	154.44	20	28.3	33.40	20
**105.40**	**119.22**	**168**	**22**	**30**	**-**	**22**
121.19	148.87	193.59	**-**	**-**	**-**	**-**
145.67	194.22	231.44	30	-	-	-
170.15	-	264.59	35	-	-	-
196.38	-	299.63	40	-	-	-
-	-	334.03	45	-	-	-

Table S8: The balanced relationship between meat intake and physical activity (cancer mortality).

Cancer mortality						
Red meat(g/day)	Processed meat(g/day)	Mixed meat(g/day)	Increased risk of mortality(%)	MSA(mins/ week)	MSA(mins/ week)	Reduced risk of mortality (%)
0	0	0	0	0	139.06	0
44.57	19.39	54.42	5	14.28	114.28	5
70.85	45.56	92.87	10	25.36	90.23	10
**88.35**	**69.70**	**117.34**	**13**	**30.00**	**-**	**13**
109.36	139.61	138.33	15			
144.37	-	180.31	18			
168.87	-	218.82	20			

Table S9: The balanced relationship between meat intake and physical activity.

	Increased risk of mortality(%)				Reduced risk of mortality(%)			
	mortality	Red meat intake (g/day)	Processed meat intake (g/day)	Mixed meat intake (g/day)	mortality	Daily steps (steps/day)	MSA (mins/week)	MSA (mins/week)
All-cause	0	0	0	0	0	1895	0	145.27
	5	32.38	13.73	25.44	5	2394.99	8.04	126.47
	10	53.30	21.56	49.57	10	2975.67	16.5	107.67
	15	74.07	30.93	73.71	15	3548.90	25.6	90.58
	**20**	**103.40**	**50**	**112.50**	**20**	**4100.00**	**39.5**	**61.52**
	25	128.23	58.13	139.27	25	4777.27	-	-
	30	163.18	82.53	191.08	30	5432.40	-	-
	35	196.32	106.94	325.94	35	6087.53	-	-
	40	-	129.60	-	40	6824.55	-	-
	45	-	154.01	-	45	7971.02	-	-
	**50**	**-**	**178.42**	**-**	**50**	**11815.00**	**-**	**-**
CVD	0	0	0	0	0	-	0	131.76
	5	43.44	37.41	34.83	5	-	7.15	92.07
	10	59.18	51.28	72.49	10	-	14.30	72.66
	15	76.67	72.13	113.83	15	-	21.30	52.96
	20	97.66	105.38	154.44	20	-	28.3	33.40
	**22**	**105.40**	**119.22**	**168**	**22**	**--**	30	-
	**25**	**121.19**	**148.87**	**193.59**	**-**	**-**	**-**	**-**
Cancer	0	0	0	0	0	-	0	148.12
	5	44.57	19.39	54.42	5	-	13.13	133.13
	10	70.85	45.56	92.87	10	-	20.19	114.06
	**13**	**88.35**	**69.70**	**117.34**	**13**	**-**	**30**	**-**
	15	109.36	139.61	138.33				

	20	144.37	-	180.31				-

Table S10: The pooled estimates of red and processed meat, physical activity and all-cause, cardiovascular disease (CVD) and cancer mortality.

Meat intake, physical activity and mortality	No. of Papers (Participants)	ES(95%CI)	P_{ES}	I^2(%)	Egger's (*P*-value)	Begg's (*P*-value)	GREAD Certainty of Evidence
**Red meat and mortality**							
All-cause mortality	18	1.12(1.04-1.20)	＜0.01	92%	0.069	0.430	Very low<br>Downgraded due to inconsistency
CVD mortality	13	1.14(1.03-1.26)	＜0.01	85%	0.208	0.596	Very low<br>Downgraded due to inconsistency
Cancer mortality	16	1.07(1.01-1.13)	＜0.01	77%	0.167	0.695	Very low<br>Downgraded due to inconsistency
**Processed meat and mortality**							
All-cause mortality	16	1.17(1.11-1.22)	＜0.01	80%	0.638	0.576	Very low<br>Downgraded due to inconsistency
CVD mortality	10	1.21(1.08-1.35)	＜0.01	83%	0.428	0.760	Very low<br>Downgraded due to inconsistency
Cancer mortality	15	1.11(1.09-1.14)	0.43	2%	0.759	0.675	low
**Mixed meat and mortality**							
All-cause mortality	10	1.22(1.15-1.30)	＜0.01	86%	0.85	0.876	Very low<br>Downgraded due to inconsistency
CVD mortality	8	1.30(1.20-1.41)	＜0.01	67%	0.485	0.917	Very low<br>Downgraded due to inconsistency

Cancer mortality	8	1.15(1.12-1.19)	0.08	46%	0.159	0.037	Very low<br>Downgraded due to publication
**muscle-strengthening activity and mortality**							
All-cause mortality	8(712617)	0.91(0.88-0.98)	＜0.01	62%	0.994	0.902	Very low<br>Downgraded due to inconsistency
CVD mortality	7(207512)	0.88(0.81-0.95)	0.11	43%	0.380	1	low
Cancer mortality	7(508807)	0.85(0.76-0.94)	0.04	54%	0.763	1	Very low<br>Downgraded due to inconsistency
**Daily steps and mortality**							
All-cause mortality	11(177297)	0.58(0.45-0.74)	＜0.01	93%	0.040	0.815	Very low<br>Downgraded due to inconsistency and publication

GRADE = Grading of Recommendations Assessment, Development and Evaluation.

PRISMA 2020 Checklist

Supplementary Table S11 PRISMA Checklist of the systematic review and meta-analysis

Section and Topic	Item #	Checklist item	Location where item is reported
**TITLE**			
Title	1	Identify the report as a systematic review.	P1
**ABSTRACT**			
Abstract	2	See the PRISMA 2020 for Abstracts checklist.	P2-3
**INTRODUCTION**			
Rationale	3	Describe the rationale for the review in the context of existing knowledge.	P4-5
Objectives	4	Provide an explicit statement of the objective(s) or question(s) the review addresses.	P4-5
**METHODS**			
Eligibility criteria	5	Specify the inclusion and exclusion criteria for the review and how studies were grouped for the syntheses.	P5-6
Information sources	6	Specify all databases, registers, websites, organisations, reference lists and other sources searched or consulted to identify studies. Specify the date when each source was last searched or consulted.	P5
Search strategy	7	Present the full search strategies for all databases, registers and websites, including any filters and limits used.	P5, Figure 1, Table S1
Selection process	8	Specify the methods used to decide whether a study met the inclusion criteria of the review, including how many reviewers screened each record and each report retrieved, whether they worked independently, and if applicable, details of automation tools used in the process.	P5-6, Figure 1
Data collection process	9	Specify the methods used to collect data from reports, including how many reviewers collected data from each report, whether they worked independently, any processes for obtaining or confirming data from study investigators, and if applicable, details of automation tools used in the process.	P5-6
Data items	10a	List and define all outcomes for which data were sought. Specify whether all results that were compatible with each outcome domain in each study were sought (e.g. for all measures, time points, analyses), and if not, the methods used to decide which results to collect.	P6
	10b	List and define all other variables for which data were sought (e.g. participant and intervention characteristics, funding sources). Describe any assumptions made about any missing or unclear information.	P6-7
Study risk of bias assessment	11	Specify the methods used to assess risk of bias in the included studies, including details of the tool(s) used, how many reviewers assessed each study and whether they worked independently, and if applicable, details of automation tools used in the process.	P6
Effect measures	12	Specify for each outcome the effect measure(s) (e.g. risk ratio, mean difference) used in the synthesis or presentation of results.	P6
Synthesis methods	13a	Describe the processes used to decide which studies were eligible for each synthesis (e.g. tabulating the study intervention characteristics and comparing against the planned groups for each synthesis (item #5)).	P5
	13b	Describe any methods required to prepare the data for presentation or synthesis, such as handling of missing summary statistics, or data conversions.	P6-7

Section and Topic	Item #	Checklist item	Location where item is reported
	13c	Describe any methods used to tabulate or visually display results of individual studies and syntheses.	P6-7
	13d	Describe any methods used to synthesize results and provide a rationale for the choice(s). If meta-analysis was performed, describe the model(s), method(s) to identify the presence and extent of statistical heterogeneity, and software package(s) used.	P6-7
	13e	Describe any methods used to explore possible causes of heterogeneity among study results (e.g. subgroup analysis, meta-regression).	P6-7
	13f	Describe any sensitivity analyses conducted to assess robustness of the synthesized results.	P6
Reporting bias assessment	14	Describe any methods used to assess risk of bias due to missing results in a synthesis (arising from reporting biases).	P6
Certainty assessment	15	Describe any methods used to assess certainty (or confidence) in the body of evidence for an outcome.	P6
**RESULTS**			
Study selection	16a	Describe the results of the search and selection process, from the number of records identified in the search to the number of studies included in the review, ideally using a flow diagram.	Figure 1
	16b	Cite studies that might appear to meet the inclusion criteria, but which were excluded, and explain why they were excluded.	Figure 1
Study characteristics	17	Cite each included study and present its characteristics.	Table 1-3, Table S2-S3, Supplementary Results P3
Risk of bias in studies	18	Present assessments of risk of bias for each included study.	P7, Table S4
Results of individual studies	19	For all outcomes, present, for each study: (a) summary statistics for each group (where appropriate) and (b) an effect estimate and its precision (e.g. confidence/credible interval), ideally using structured tables or plots.	Figure 2-3, Figure S1-S3,
Results of syntheses	20a	For each synthesis, briefly summarise the characteristics and risk of bias among contributing studies.	Supplementary Results P3-5
	20b	Present results of all statistical syntheses conducted. If meta-analysis was done, present for each the summary estimate and its precision (e.g. confidence/credible interval) and measures of statistical heterogeneity. If comparing groups, describe the direction of the effect.	Supplementary Results P3-5, Figure 2-3, Figure S1-S3
	20c	Present results of all investigations of possible causes of heterogeneity among study results.	Supplementary Results P3-5, P10-11, Table S5
	20d	Present results of all sensitivity analyses conducted to assess the robustness of the synthesized results.	Figure S9-S13
Reporting biases	21	Present assessments of risk of bias due to missing results (arising from reporting biases) for each synthesis assessed.	Supplementary Results P3-5
Certainty of evidence	22	Present assessments of certainty (or confidence) in the body of evidence for each outcome assessed.	P11, Figure S10
**DISCUSSION**			
Discussion	23a	Provide a general interpretation of the results in the context of other evidence.	P112-15
	23b	Discuss any limitations of the evidence included in the review.	P15-16

Section and Topic	Item #	Checklist item	Location where item is reported
	23c	Discuss any limitations of the review processes used.	P15-16
	23d	Discuss implications of the results for practice, policy, and future research.	P16
**OTHER INFORMATION**			
Registration and protocol	24a	Provide registration information for the review, including register name and registration number, or state that the review was not registered.	P5
	24b	Indicate where the review protocol can be accessed, or state that a protocol was not prepared.	P5
	24c	Describe and explain any amendments to information provided at registration or in the protocol.	NA
Support	25	Describe sources of financial or non-financial support for the review, and the role of the funders or sponsors in the review.	P3
Competing interests	26	Declare any competing interests of review authors.	P16
Availability of data, code and other materials	27	Report which of the following are publicly available and where they can be found: template data collection forms; data extracted from included studies; data used for all analyses; analytic code; any other materials used in the review.	Table 1-3, Table S2-S3,

From: Page MJ, McKenzie JE, Bossuyt PM, Boutron I, Hoffmann TC, Mulrow CD, et al. The PRISMA 2020 statement: an updated guideline for reporting systematic reviews. BMJ 2021;372:n71. doi: 10.1136/bmj.n71

For more information, visit: http://www.prisma-statement.org/

PRISMA 2020 for Abstracts Checklist

Supplementary Table S12 PRISMA Abstracts Checklist of the systematic review and meta-analysis

Section and Topic	Item #	Checklist item	Reported (Yes/No)
**TITLE**			
Title	1	Identify the report as a systematic review.	Yes
**BACKGRO UND**			
Objectives	2	Provide an explicit statement of the main objective(s) or question(s) the review addresses.	Yes
**METHO DS**			
Eligibility criteria	3	Specify the inclusion and exclusion criteria for the review.	Yes
Information sources	4	Specify the information sources (e.g. databases, registers) used to identify studies and the date when each was last searched.	Yes
Risk of bias	5	Specify the methods used to assess risk of bias in the included studies.	Yes
Synthesis of results	6	Specify the methods used to present and synthesise results.	Yes
**RESULTS**			
Included studies	7	Give the total number of included studies and participants and summarise relevant characteristics of studies.	Yes
Synthesis of results	8	Present results for main outcomes, preferably indicating the number of included studies and participants for each. If meta-analysis was done, report the summary estimate and confidence/credible interval. If comparing groups, indicate the direction of the effect (i.e. which group is fav oured).	Yes
**DISCUSSION**			
Limitations of evidence	9	Provide a brief summary of the limitations of the evidence included in the review (e.g. study risk of bias, inconsistency and imprecision).	Yes
Interpretation	10	Provide a general interpretation of the results and important implications.	Yes
**O THER**			
Funding	11	Specify the primary source of funding for the review.	Yes
Registration	12	Provide the register name and registration number.	Yes

Supplementary Table S13: Number of studies from different databases.

**Meat intake**		**MSA**		**Daily steps**	
**databases**	**the number of articles**	**databases**	**the number of articles**	**databases**	**the number of articles**
ARIC study, CARDIA study, CHS, FHS, FOS, and MESA	1	NHIS	3	Womenˈs Health Study (WHS)	1
Netherlands Cohort Study	1	CHAMP	1	a nation-wide multicenter physical activity surveillance study in Norway	1
The Nurses' Health Study The Health Professionals' Followup Study	2	NHANES	1	Toledo Study for Healthy Aging (TSHA)	1
AHS-2	1	CPSIINC	1	British Regional Heart Study	1
The Danish National Survey on Diet and Physical Activity	1	The Aerobics Center Longitudinal Study	1	elderly people living in Niigata City, Japan	1
UK Biobank	2	Womenˈs Health Study	1	The AUSDIAB study	1
The SUN project	1	HSE/SHS	1	Coronary Artery Risk Development in Young Adults (CARDIA) study	1
NIH-AARP Diet and Health Study	1			NHANES	1
BWHS	1			The ActiFE Ulm study	1
JPHC study	1			(CPS)-II Nutrition Cohort	1
EPIC	1			HCS	1
the NIH-AARP Diet and Health Study.	1				
SWHS and SMHS	1				
NIPPON DATA80	1				
COSM and SMC	1				
NHIS	1				
NHANES III	1				

8 prospective cohort studies from Bangladesh, mainland China, Japan, Korea, and Taiwan were included	1				
OXCHECK study	1				
a mailed questionnaire for the ACS′s2 CPS II	2				
The Golestan Cohort Study	1				
The Minnesota Colon Cancer Control Study	1				
PURE Study	1				
The WHI study	1				

Supplementary Figures

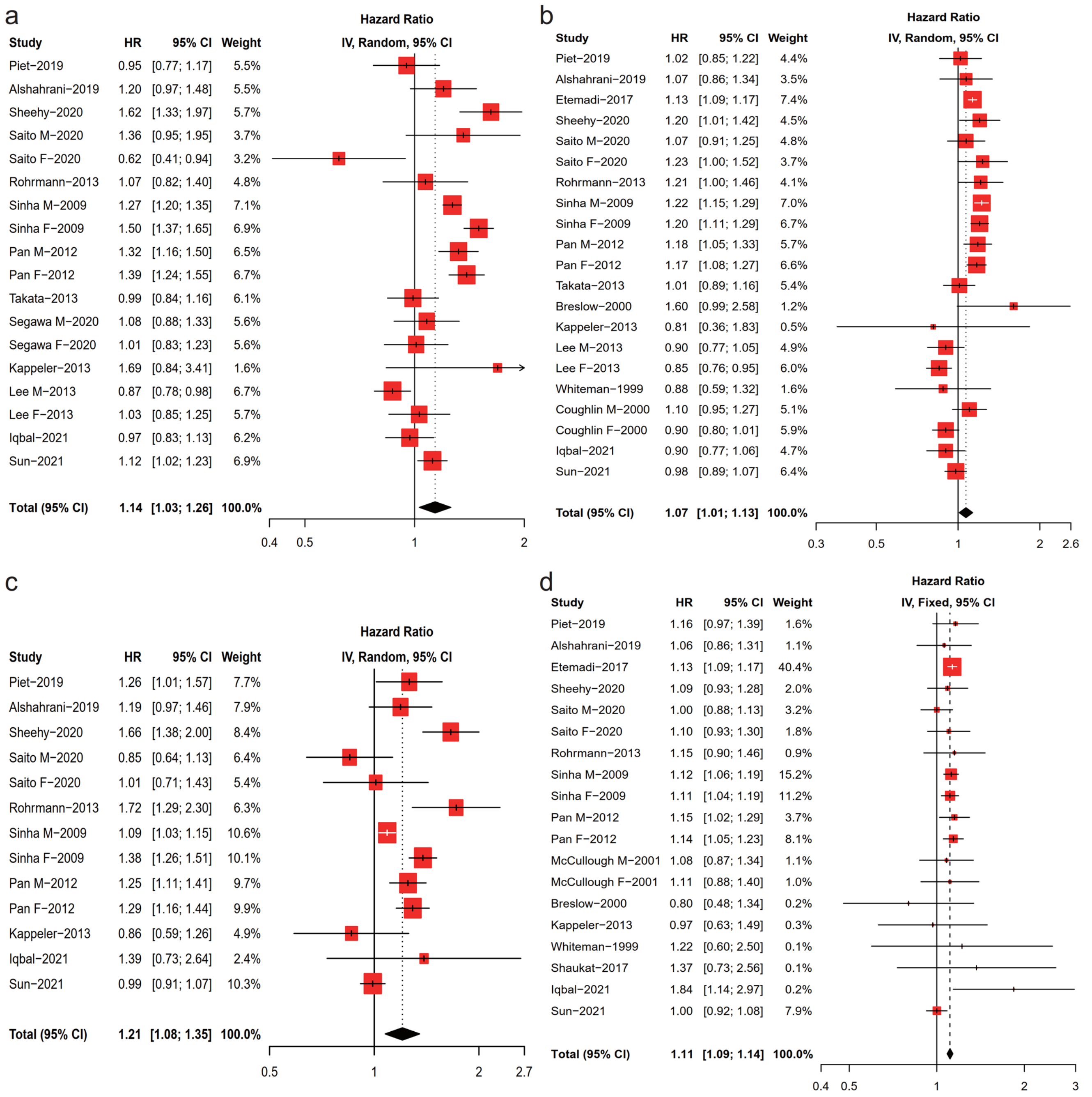

Figure S1. Forest plots of meta-analyses of red and processed meat intake and mortality risk. a) red meat intake and CVD mortality; b) red meat intake and cancer mortality; c) processed meat intake and CVD mortality; d) processed meat intake and cancer mortality.

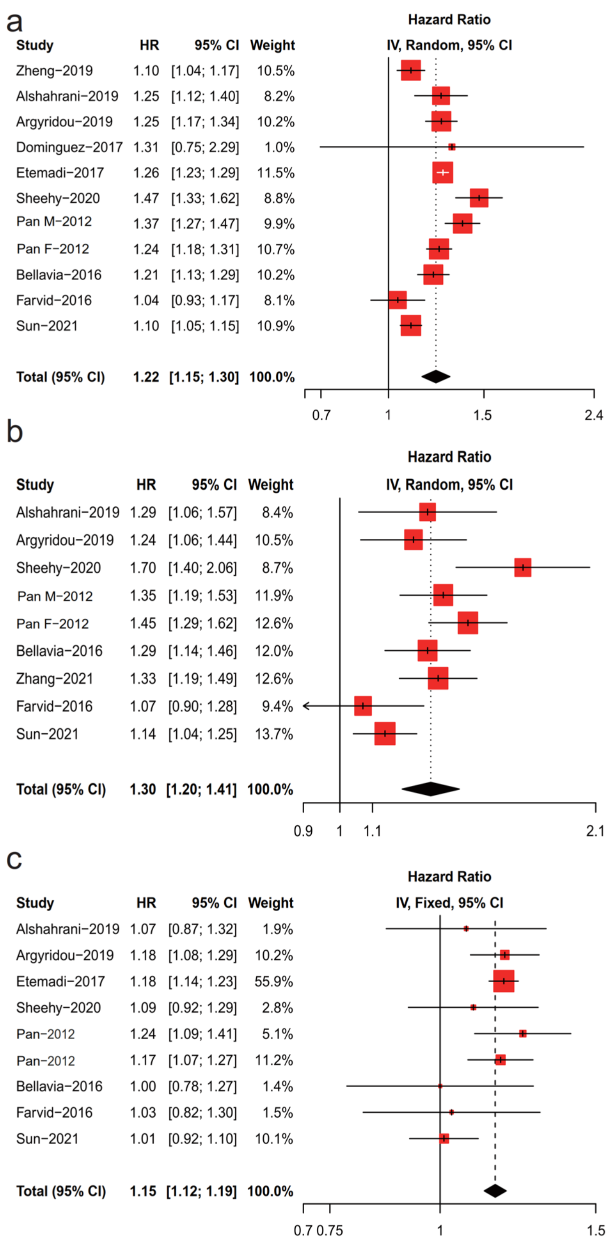

Figure S2. Forest plots of meta-analyses of mixed intake of red and processed meat and mortality risk. a) mixed intake of red and processed meat and all-cause mortality; b) mixed intake of red and processed meat and CVD mortality; c) mixed intake of red and processed meat and cancer mortality.

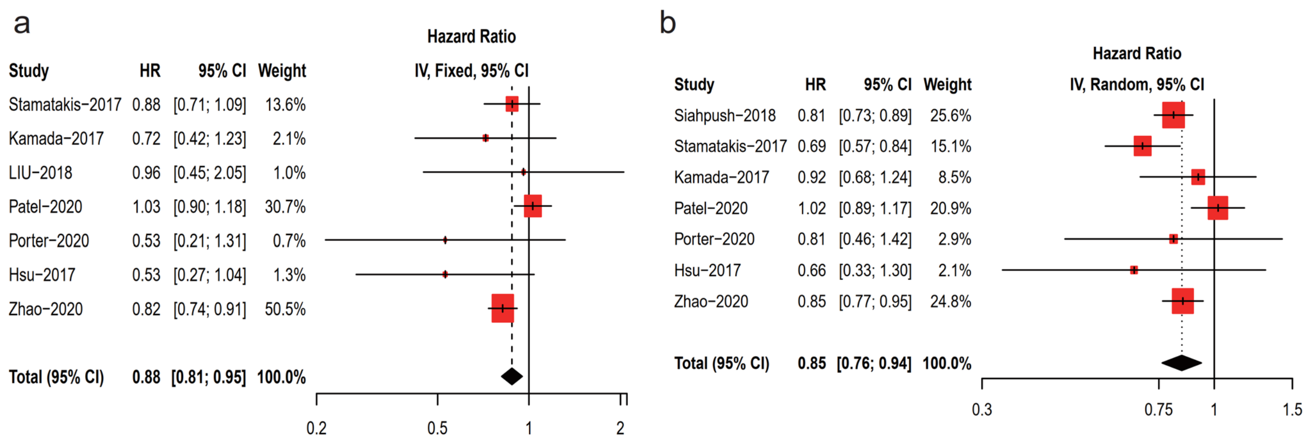

Figure S3. Forest plots of meta-analyses of muscle-strengthening activity (MSA) and mortality outcomes. a) MSA and CVD mortality; b) MSA and cancer mortality.

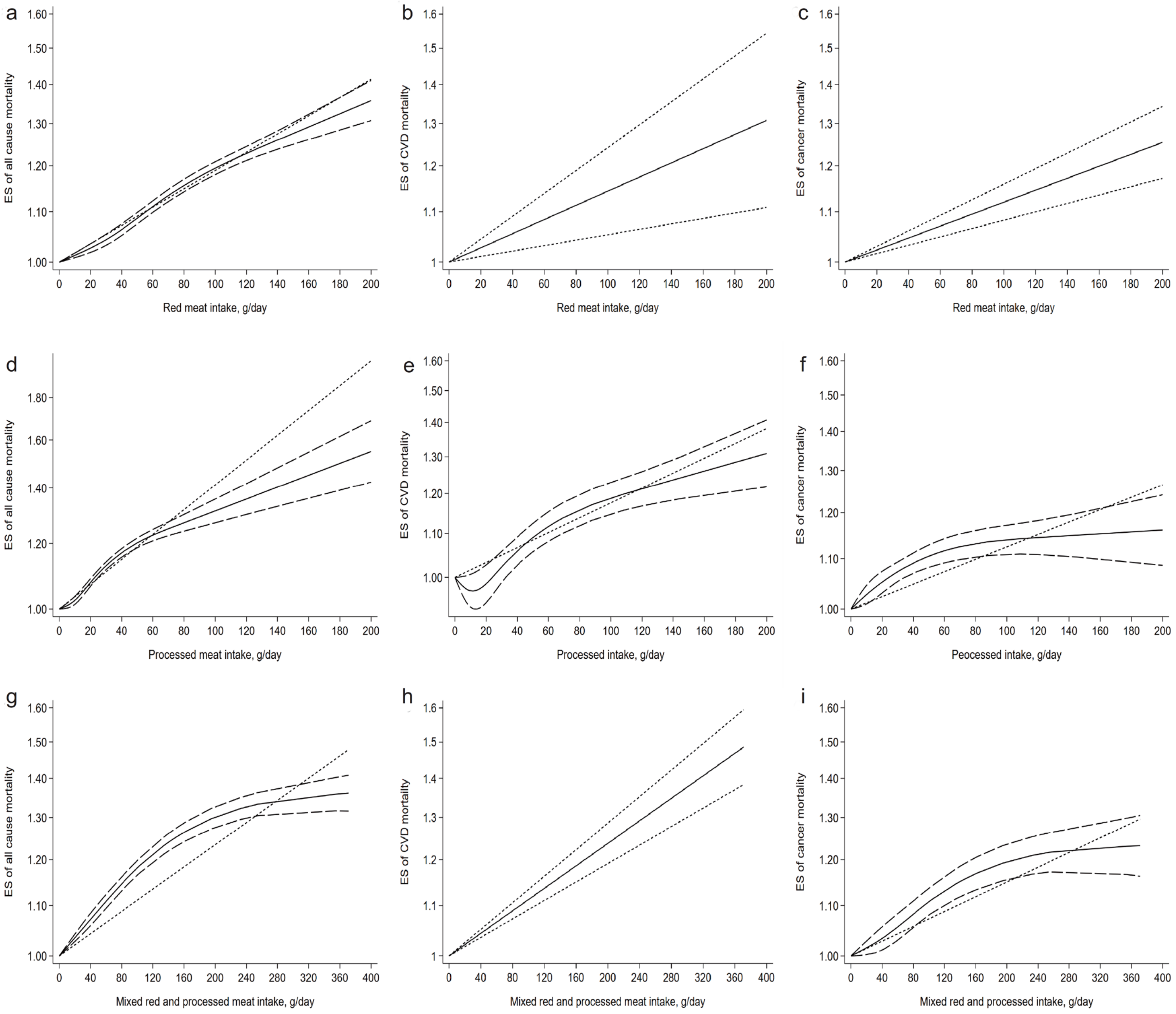

Figure S4: Linear and non-linear dose-response relationships between red and processed meat intake and mortality risk. a) red meat intake and all-cause mortality; b) red meat intake and CVD mortality; c) red meat intake and cancer mortality; d) processed meat intake and all-cause mortality; e) processed meat intake and CVD mortality; f) processed meat intake and cancer mortality; g) mixed meat intake and all-cause mortality; h) mixed meat intake and CVD mortality; i) mixed meat intake and cancer mortality.

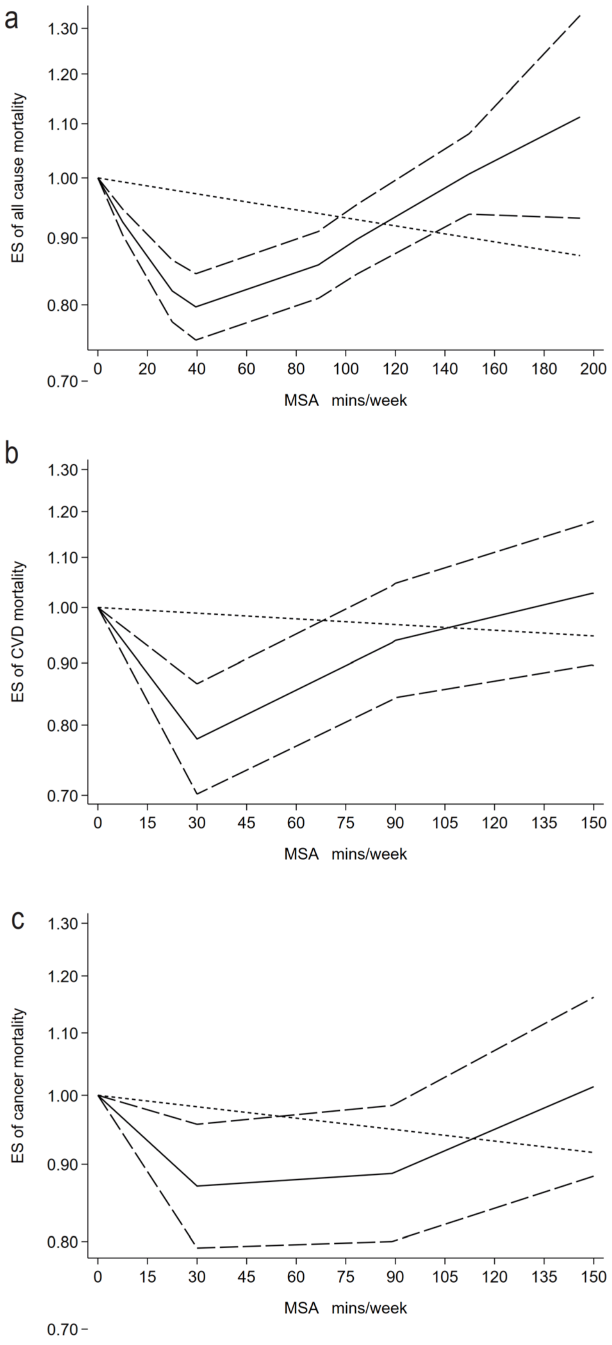

Figure S5. Non-linear dose-response relationships between MSA and mortality risk. a) MSA and all-cause mortality; b) MSA and CVD mortality; c) MSA and cancer mortality.

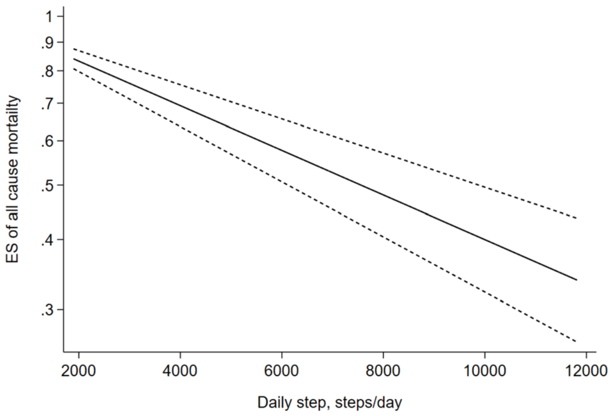

Figure S6. Linear dose-response relationships between daily steps all-cause mortality.

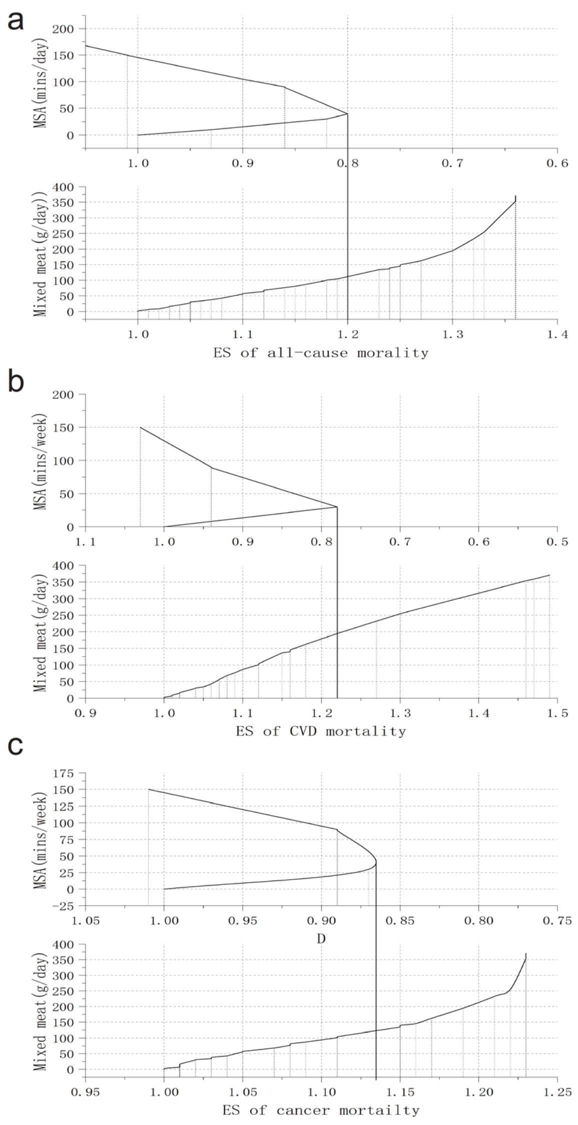

Figure S7. Balanced associations between mixed intake of red and processed meat and muscle-strengthening activity (MSA). a) mixed intake of red and processed meat, MSA and all-cause mortality; b) mixed intake of red and processed meat, MSA and CVD mortality; c) mixed intake of red and processed meat, MSA and cancer mortality.

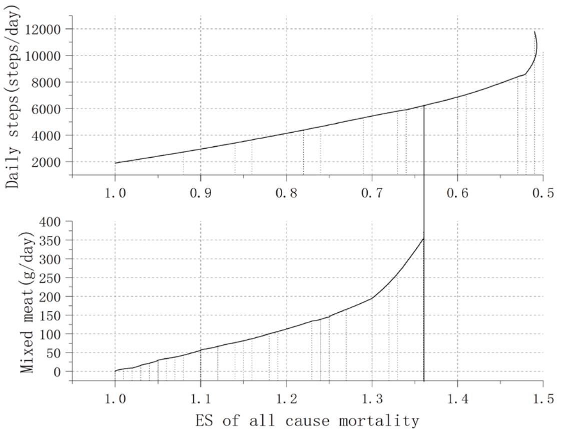

Figure S8. Balanced associations between mixed intake red and processed meat, daily steps and all-cause mortality.

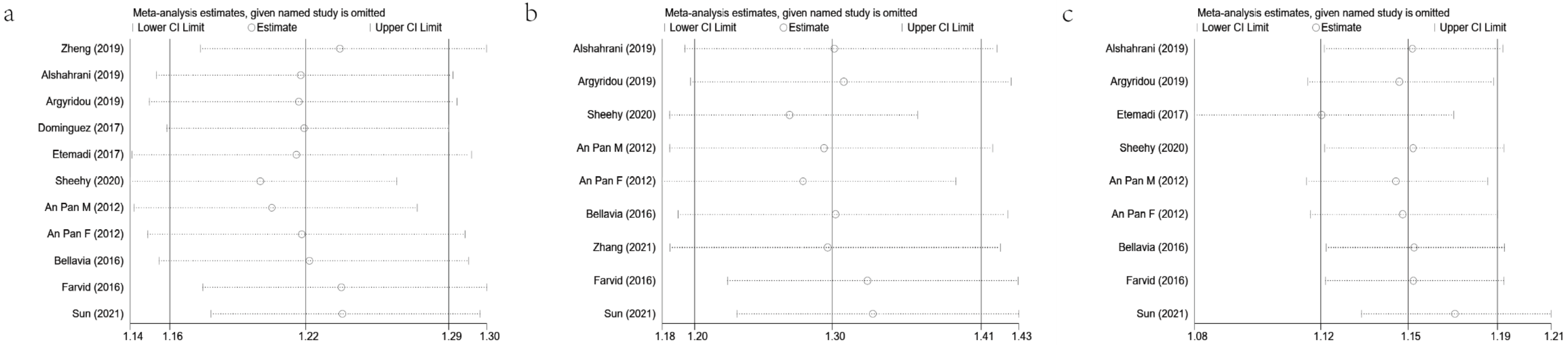

Figure S9. Sensitivity analysis of mixed red and processed intake and mortality outcomes.

a) mixed intake of red and processed meat and all-cause mortality; b) mixed intake of red and processed meat and CVD mortality; c) mixed intake of red and processed meat and cancer mortality.

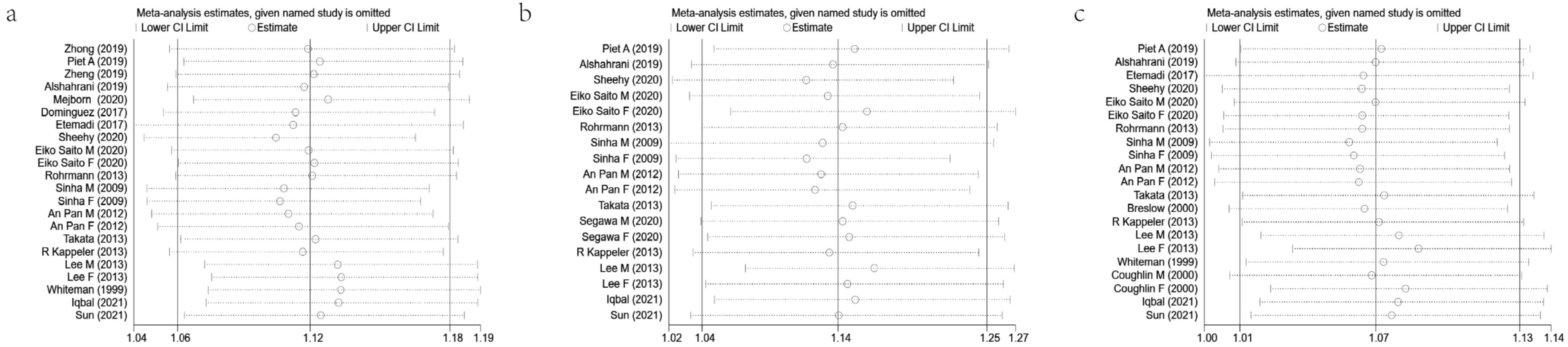

Figure S10. Sensitivity analysis of red meat intake and mortality outcomes.

a) intake of red meat and all-cause mortality; b) intake of red meat and CVD mortality; c) intake of red meat and cancer mortality.

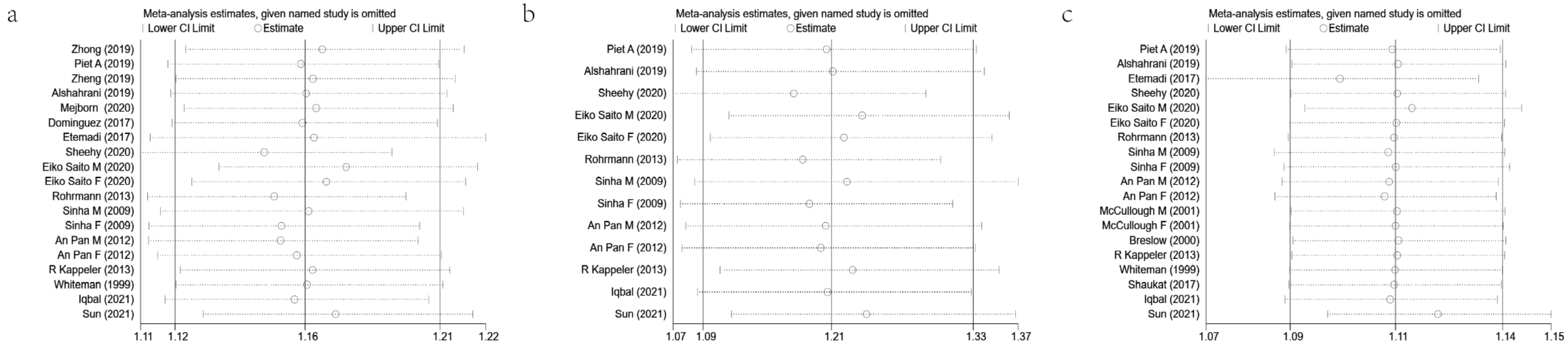

Figure S11. Sensitivity analysis of processed intake and mortality outcomes.

a) intake of processed meat and all-cause mortality; b) intake of processed meat and CVD mortality; c) intake of processed meat and cancer mortality.

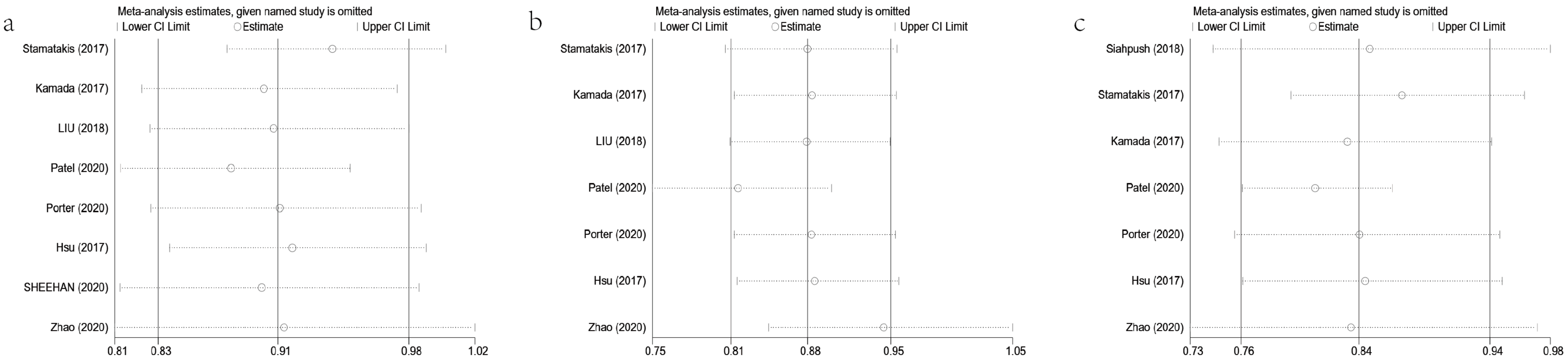

Figure S12. Sensitivity analysis of muscle-strengthening activity (MSA) and mortality outcomes.

a) MSA and all-cause mortality; b) MSA and processed meat and CVD mortality; c) MSA and cancer mortality.

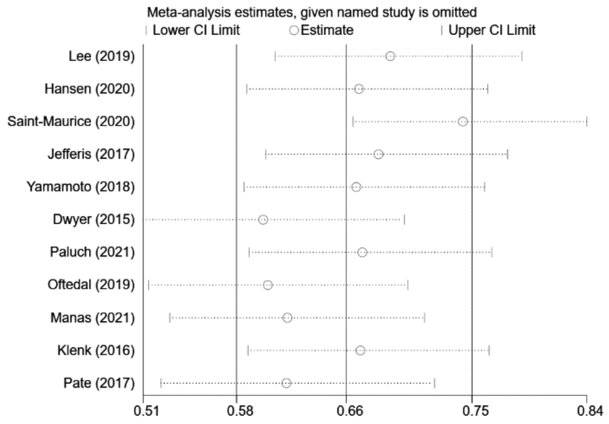

Figure S13. Sensitivity analysis of daily steps and all-cause mortality.

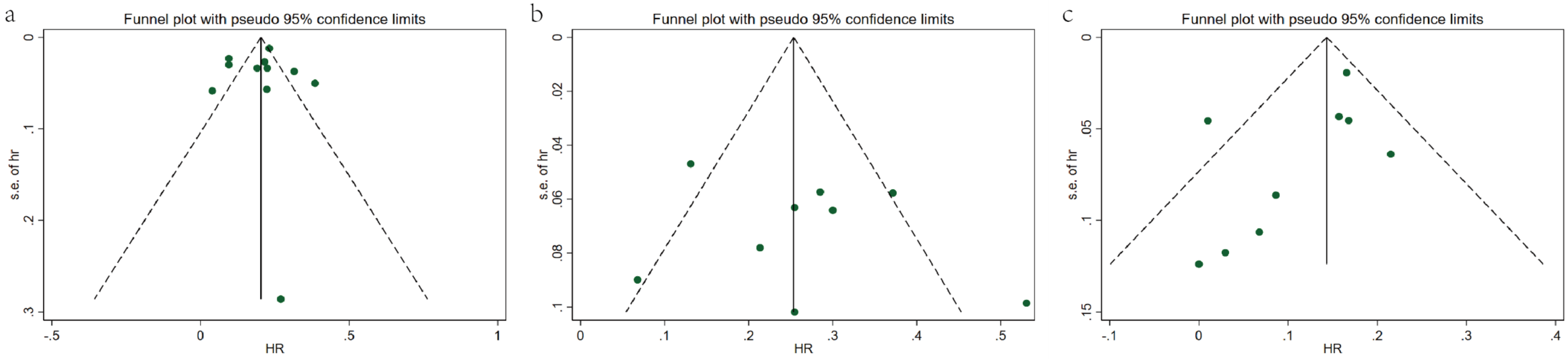

Figure S14. Funnel plot of included studies in the meta-analysis of the intake of mixed red and processed meat and mortality from all-cause, CVD, and cancer.

a) mixed intake of red and processed meat and all-cause mortality; b) mixed intake of red and processed meat and CVD mortality; c) mixed intake of red and processed meat and cancer mortality.

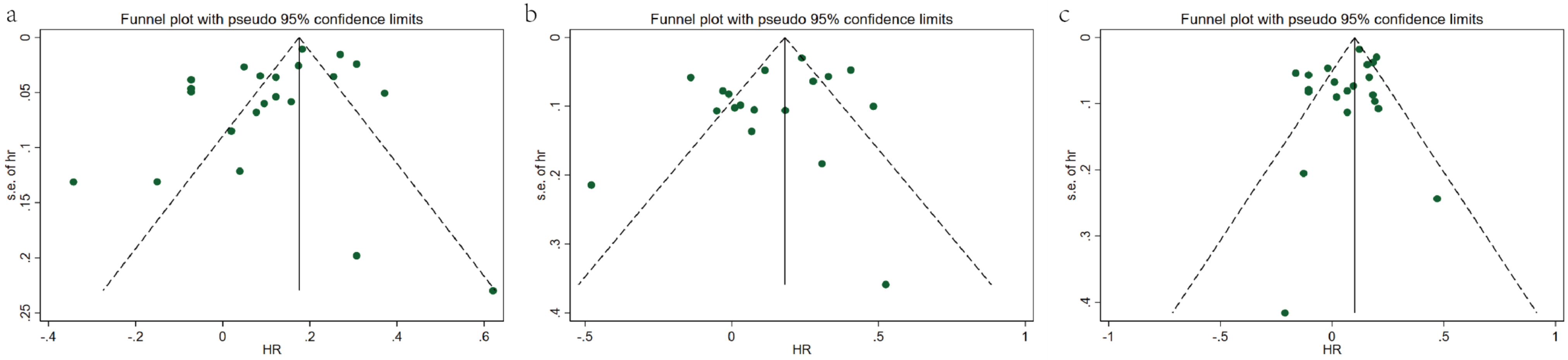

Figure S15. Funnel plot of included studies in the meta-analysis of the intake of red meat and mortality from all-cause, CVD, and cancer.

a) red meat intake and all-cause mortality; b) red meat intake and CVD mortality; c) red meat intake and cancer mortality.

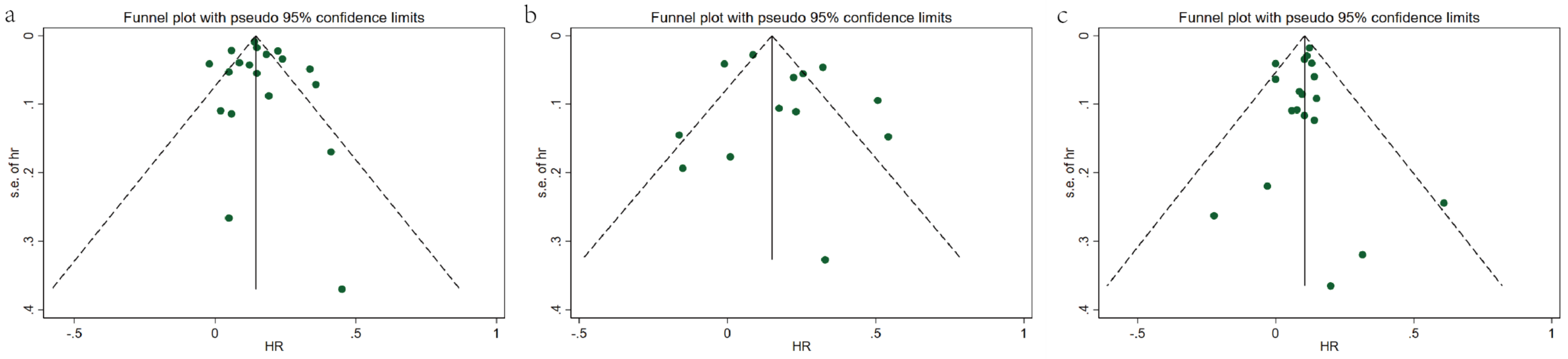

Figure S16. Funnel plot of included studies in the meta-analysis of the intake of processed meat and mortality from all-cause, CVD, and cancer.

a) processed meat intake and all-cause mortality; b) processed meat intake and CVD mortality; c) processed meat intake and cancer mortality.

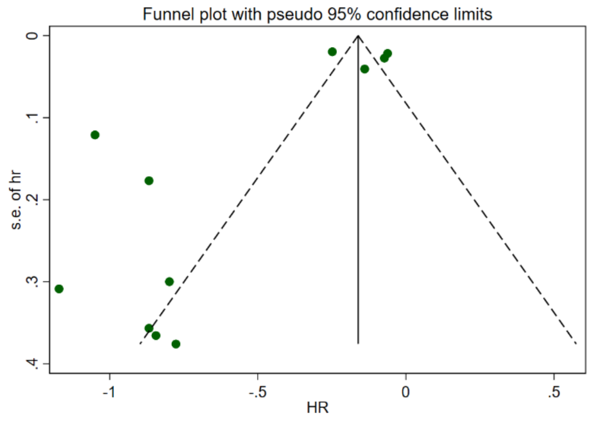

Figure S17. Funnel plot of included studies in the meta-analysis of daily steps and all-cause mortality.
